# Metagenome Sequence Analysis of Filamentous Microbial Communities Obtained from Geochemically Distinct Geothermal Channels Reveals Specialization of Three Aquificales Lineages

**DOI:** 10.3389/fmicb.2013.00084

**Published:** 2013-05-29

**Authors:** Cristina Takacs-Vesbach, William P. Inskeep, Zackary J. Jay, Markus J. Herrgard, Douglas B. Rusch, Susannah G. Tringe, Mark A. Kozubal, Natsuko Hamamura, Richard E. Macur, Bruce W. Fouke, Anna-Louise Reysenbach, Timothy R. McDermott, Ryan deM. Jennings, Nicolas W. Hengartner, Gary Xie

**Affiliations:** ^1^Department of Biology, University of New MexicoAlbuquerque, NM, USA; ^2^Department of Land Resources and Environmental Sciences, Thermal Biology Institute, Montana State UniversityBozeman, MT, USA; ^3^Novo Nordisk Foundation Center for Biosustainability, Technical University of DenmarkHørsholm, Denmark; ^4^Center for Genomics and Bioinformatics, Indiana UniversityBloomington, IN, USA; ^5^Department of Energy-Joint Genome InstituteWalnut Creek, CA, USA; ^6^Center for Marine Environmental Studies, Ehime UniversityMatsuyama, Ehime, Japan; ^7^Roy J. Carver Biotechnology Center, University of IllinoisUrbana, IL, USA; ^8^Department of Biology, Portland State UniversityPortland, OR, USA; ^9^Computer, Computational, and Statistical Sciences Division, Los Alamos National LaboratoryLos Alamos, NM, USA; ^10^Bioscience Division, Los Alamos National LaboratoryLos Alamos, NM, USA

**Keywords:** thermophiles, functional genomics, phylogeny, autotrophic processes, sulfide oxidation

## Abstract

The Aquificales are thermophilic microorganisms that inhabit hydrothermal systems worldwide and are considered one of the earliest lineages of the domain *Bacteria*. We analyzed metagenome sequence obtained from six thermal “filamentous streamer” communities (∼40 Mbp per site), which targeted three different groups of Aquificales found in Yellowstone National Park (YNP). Unassembled metagenome sequence and PCR-amplified 16S rRNA gene libraries revealed that acidic, sulfidic sites were dominated by *Hydrogenobaculum* (Aquificaceae) populations, whereas the circum-neutral pH (6.5–7.8) sites containing dissolved sulfide were dominated by *Sulfurihydrogenibium* spp. (Hydrogenothermaceae). *Thermocrinis* (Aquificaceae) populations were found primarily in the circum-neutral sites with undetectable sulfide, and to a lesser extent in one sulfidic system at pH 8. Phylogenetic analysis of assembled sequence containing 16S rRNA genes as well as conserved protein-encoding genes revealed that the composition and function of these communities varied across geochemical conditions. Each Aquificales lineage contained genes for CO_2_ fixation by the reverse-TCA cycle, but only the *Sulfurihydrogenibium* populations perform citrate cleavage using ATP citrate lyase (Acl). The Aquificaceae populations use an alternative pathway catalyzed by two separate enzymes, citryl-CoA synthetase (Ccs), and citryl-CoA lyase (Ccl). All three Aquificales lineages contained evidence of aerobic respiration, albeit due to completely different types of heme Cu oxidases (subunit I) involved in oxygen reduction. The distribution of Aquificales populations and differences among functional genes involved in energy generation and electron transport is consistent with the hypothesis that geochemical parameters (e.g., pH, sulfide, H_2_, O_2_) have resulted in niche specialization among members of the Aquificales.

## Introduction

The order Aquificales represents a group of thermophilic microorganisms that inhabit marine and terrestrial hydrothermal systems worldwide (Ferrera et al., 2007). This lineage is of significant interest because its members are believed to comprise the deepest lineage of the domain *Bacteria* (Coenye and Vandamme, 2004; Barion et al., 2007), although alternative evolutionary histories have been suggested (Griffiths and Gupta, 2004; Boussau et al., 2008; Zhaxybayeva et al., 2009). The Aquificales include two predominant families, the Hydrogenothermaceae and Aquificaceae, and both are well-represented in different geothermal features of Yellowstone National Park (YNP) (Reysenbach et al., 2005). Members of the Hydrogenothermaceae include the genus *Sulfurihydrogenibium*, which inhabit circum-neutral sulfidic springs in YNP (Hugenholtz et al., 1998; Reysenbach et al., 2000b, 2005). The Aquificaceae comprise two divergent groups: *Hydrogenobaculum* spp. are found predominantly in low-pH systems (pH < 4) (Jackson et al., 2001; Langner et al., 2001; Macur et al., 2004; D’Imperio et al., 2007, 2008; Hamamura et al., 2009), while *Thermocrinis-*like organisms generally exhibit higher-pH ranges (pH 6–9), overlapping with the near circum-neutral optimum for *Sulfurihydrogenibium* (Reysenbach et al., 1994; Blank et al., 2002).

Given our limited understanding of their phylogenetic history and metabolic potential, numerous questions remain regarding the ecology and evolution of the Aquificales. For example, the Aquificales are important primary producers in hydrothermal systems (Harmsen et al., 1997; Yamamoto et al., 1998; Reysenbach et al., 1999, 2000a; Blank et al., 2002; Eder and Huber, 2002; Inagaki et al., 2003; Spear et al., 2005) and several members of this lineage have been shown to use the reductive tricarboxylic acid (r-TCA) cycle for the fixation of carbon dioxide (Shiba et al., 1985; Beh et al., 1993; Ferrera et al., 2007). However, several members of the order are also capable of heterotrophy (Huber et al., 1998; Nakagawa et al., 2005; Caldwell et al., 2010), which makes their ecological role as possible primary producers unclear. Moreover, the diversity and variation of r-TCA-specific enzymes and substrates used by cultured members has led to speculation regarding the origin and the distribution of this pathway within the phylum (Hugler et al., 2007).

The Aquificales were originally named for the ability of the type strain, *Aquifex pyrophilus*, to oxidize molecular hydrogen to water (Huber et al., 1992). Consequently, hydrogen oxidation was generalized to the entire order based on the phenotype of a few cultivated members (Reysenbach and Cady, 2001; Donahoe-Christiansen et al., 2004; Huber and Eder, 2006; D’Imperio et al., 2008). Additionally, the abundance of molecular hydrogen in hydrothermal systems was taken as evidence for the predominance of this metabolism in thermal features from YNP (Spear et al., 2005). However, the recent isolation of several new terrestrial species indicates some organisms within this group are unable to grow on hydrogen and do not contain Group I Ni-Fe hydrogenases (Reysenbach et al., 2009). Diverse metabolisms have been detected among cultured members of the Aquificales including the use of H_2_, elemental sulfur, and thiosulfate as energy sources, and although generally aerobic, some members are microaerophilic and/or utilize nitrate as an electron acceptor (D’Imperio et al., 2008; Reysenbach et al., 2009). Thus, while the metabolic potential within the Aquificales is largely known from a few well-studied isolates, there is not an equal distribution of representatives from each of the families in culture, and their physiological capabilities remain unknown, especially under the environmental conditions that are characteristic of their natural habitats.

Much of what is known about the distribution of the Aquificales is based on molecular diversity studies across different habitat types (Reysenbach et al., 1994, 2000b; Hugenholtz et al., 1998; Stohr et al., 2001; Van Dover et al., 2001; Inagaki et al., 2003), although their ecology has been inferred largely from the geochemical conditions they inhabit (Fouke et al., 2000; Spear et al., 2005; Hall et al., 2008; Hamamura et al., 2009) and the physiology of the few cultivated members (Jahnke et al., 2001; Takai et al., 2002; Reysenbach et al., 2009). Based on molecular diversity surveys of 16S rRNA and metabolic genes, different Aquificales lineages do not generally share the same habitats in YNP (Reysenbach et al., 2005), although exceptions have been noted where *Thermocrinis-* and *Sulfurihydrogenibium*-like organisms have been found together in sulfidic circum-neutral pH channels (Hall et al., 2008; Hamamura et al., 2009). Therefore, the distinct separation of different Aquificales lineages as a function of geochemical conditions provides a unique opportunity to study the evolutionary and ecological history of diverse members of this group *in situ*. Here we provide a phylogenetic and functional analysis of metagenome sequence obtained from six Aquificales “streamer” communities, representing two replicate communities of each of the three major Aquificales lineages found in high-velocity outflow channels of YNP. The distribution and metabolic potential of Aquificales in these distinct habitat types was correlated with the geochemical attributes and conditions measured in these same locations.

## Results

### Environmental and geochemical context

**Figure 1:**
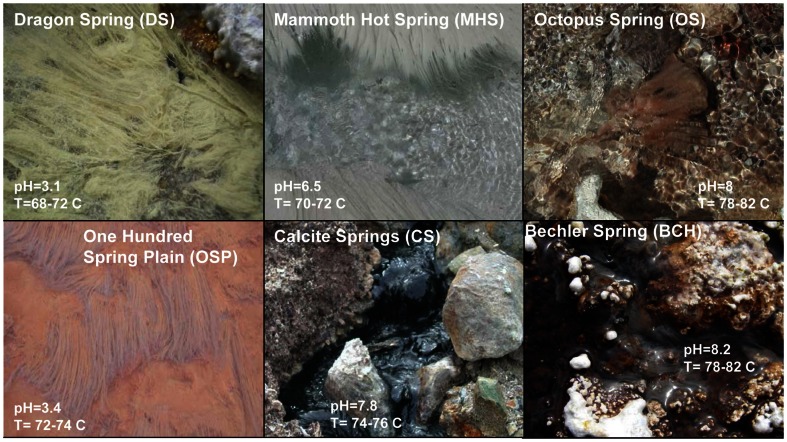
**Site photographs of Aquificales “streamer” communities sampled from Yellowstone National Park**. The sites represent diverse geochemical environments as noted from the different mineralogy apparent visually [DS_9 (elemental sulfur); OSP_14 (Fe-oxides); MHS_10 (calcium carbonate); CS_12 (pyrite, amorphous Fe-sulfides); OS_11 (none); BCH_13 (none, Fe(III)-staining on silica)].

Each high-temperature microbial community (including associated biomineralized solid-phases) was sampled from the primary flow-path (e.g., Veysey et al., 2008) of geothermal outflow channels with high velocities ranging from 0.2 to 0.5 ms^−1^. The sites were chosen to obtain a range in pH (3–8) and other geochemical attributes such as dissolved oxygen, dissolved sulfide, and/or predominant solid-phases associated with each microbial community (Figure 1; Table 1). Moreover, the physical and geochemical characteristics of these sites provide a representative subset of major Aquificales habitat types common in YNP (Reysenbach et al., 2005). Filamentous Aquificales “streamer” communities are generally found along the primary flow paths of thermal channels and colonize hydrodynamic regimes of shallow (<2 cm deep), high-velocity, turbulent (Reynold’s Number > 100,000; Fouke, 2011) spring water. These zones exhibit rapid outgassing of dissolved gases such as CO_2_ and H_2_S, and in-gassing of oxygen (Inskeep et al., 2005; Kandianis et al., 2008; Fouke, 2011).

**Table 1:** **Sample location, aqueous geochemical parameters^1^ and predominant solid-phases^2^ associated with six Aquificales communities in Yellowstone National Park (YNP)**.

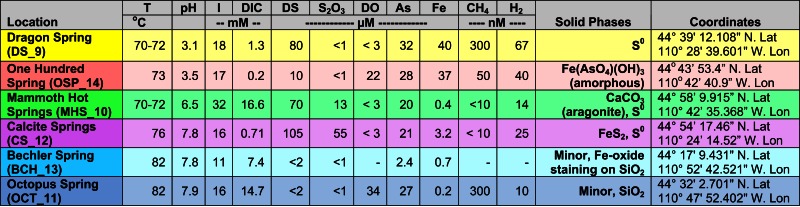

*^1^I, ionic strength calculated from aqueous geochemical modeling at sample temperature; DIC, dissolved inorganic C; DS, dissolved sulfide; S_2_O_3_, thiosulfate; DO, dissolved oxygen; CH_4_ and H_2_ values are for aqueous species*.

*^2^Predominant solid phases determined using scanning electron microscopy (FE-SEM) coupled with energy dispersive analysis of x-rays (EDAX) and x-ray diffraction (XRD)*.

The acidic (pH 3–3.5) sites in Norris Geyser Basin (NGB) represent two common Aquificales habitats in YNP. The relatively high concentration of H_2_S(aq) in *Dragon Spring* (DS_9) (80–100 μM) results in the deposition of copious amounts of elemental sulfur due to both biotic and abiotic oxidation (D’Imperio et al., 2008), whereas lower concentrations of H_2_S(aq) found in the *One Hundred Spring Plain* (OSP_14) site (<10 μM) are not sufficient to form elemental sulfur. In contrast, the “streamer” communities at OSP_14 form at a transition from reduced to oxygenated source waters and subsequent deposition of Fe-oxides (Figure 1). Scanning electron microscopy, elemental analysis, and electron diffraction confirm that elemental sulfur is the dominant solid phase in DS_9, and amorphous Fe(III)-oxide is the predominant phase associated with the community at OSP_14 (Langner et al., 2001; Inskeep et al., 2005) (Figure 1), although the gray discoloration of these Fe-oxides likely results from interaction of low concentrations of H_2_S with ferric oxides.

The outflow channels at *Mammoth Hot Springs* (MHS_10) and *Calcite Springs* (CS_12) both contain high concentrations of dissolved sulfide (>100 μM), but other geochemical differences result in different solid-phases biomineralized within the “streamer” fabric (Figure 1). The hydrothermal fluids discharging at MHS intersect the Madison Limestone formation (Fournier, 1989; Fouke, 2011) and as a result, contain high concentrations of dissolved inorganic carbon (DIC), Ca, and to a lesser extent Mg. Upon discharge, CaCO_3_ is biomineralized as aragonite needles within and along microbial filaments forming the “streamer” fabric (Figure 1) (Fouke et al., 2003; Kandianis et al., 2008; Fouke, 2011). In contrast, the black, filamentous structures at CS_12 are comprised primarily of pyrite formed along and within intertwined microbial filaments. The higher-pH, dissolved Fe, and total dissolved sulfide at *Calcite Spring* all favor the precipitation of iron sulfides relative to MHS_10 where no pyrite is observed. Rhombohedral crystals of elemental sulfur were found within the “streamers” of both MHS_10 and CS_12; however, the precipitation of elemental sulfur due to reaction of dissolved sulfide with oxygen is favored at low-pH (Xu et al., 1998; Nordstrom et al., 2005) when H_2_S(aq)/HS^−^ ≫ 1 (the pKa of H_2_S at 70°C is ∼6.8; Amend and Shock, 2001).

Higher-pH (pH 7.5–8) “streamer” communities were also sampled from the outflow channels of low sulfide, alkaline siliceous springs (*Octopus* and *Bechler Springs* (OS_11 and BCH_13). Under these conditions, filamentous communities within the flow channels at temperatures ranging from 78 to 82°C appear light-pink to gelatinous, and exhibit no obvious deposition of reaction products that may have formed from the oxidation of reduced species (e.g., no Fe-oxides, elemental sulfur or pyrite solid-phases are formed). This is consistent with the geochemical characteristics of these systems indicating low concentrations of reduced species such as sulfide, hydrogen and Fe(II), and mainly comprised of dissolved Na, Cl, and Si (Table 1). The predominant solid-phase(s) deposited along the evaporative margins of these channels are various forms of silica (although Fe-oxide staining is evident in BCH_13).

### Taxonomic classification of metagenome sequence

**Figure 2:**
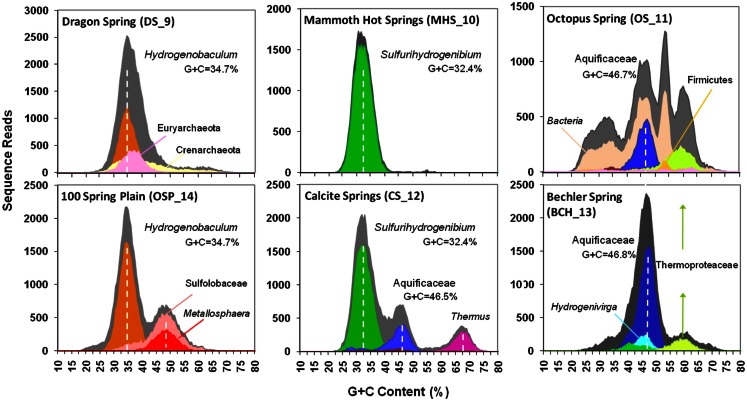
**Frequency plots of G + C content (%) of random shotgun sequence reads obtained from six Aquificales habitats in Yellowstone National Park (YNP)**. Phylogenetic classification of each sequence read (∼800 bp) was performed using MEGAN (based on “blastx”), and provides one method for visualizing the predominant phylotypes that contribute to metagenome sequence in these environments (gray = total reads; orange = *Hydrogenobaculum;* red = *Metallosphaera;* green = *Sulfurihydrogenibium;* violet = *Thermus;* blue = Aquificaceae; light-blue = *Hydrogenivirga*; light-orange = Domain *Bacteria*; light-green = Thermoproteaceae).

**Figure A1:**
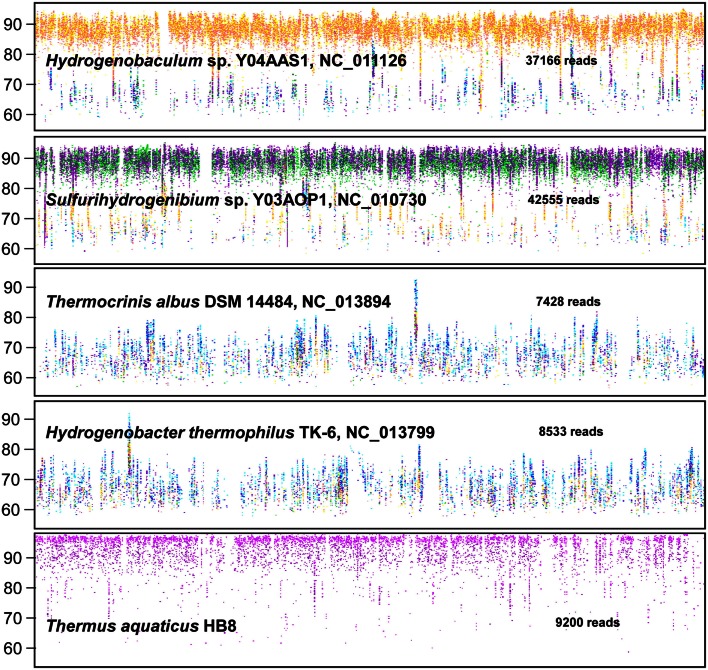
**Recruitment of metagenome sequence reads (∼800 bp per read) from six Aquificales streamer samples to the most relevant reference genomes currently available in reference databases**. Recruitment of metagenome sequence fragments across the complete genomes (*x*-axis) are shown from 50 to 100% nucleotide identity (*y*-axis). Site Colors: Yellow = *Dragon Spring* (DS_9), Red = *One Hundred Spring Plain* (OSP_14); Green = Mammoth Hot Springs (MHS_10), Violet = *Calcite Springs* (CS_12); Dark-Blue = *Octopus Spring* (OS_11), light-blue = *Bechler Springs* (BCH_13). Reference genomes: *Hydrogenobaculum* sp. Y04AAS1; *Sulfurihydrogenibium* sp. Y03AOP1 (with and without the 220 Mb of pyrosequence for MHS_10); *Thermocrinis albus* DSM 14484; *Hydrogenobacter thermophilus* TK-6; *Thermus aquaticus* HB8 (Plot constructed using JCVI bioinformatic utilities, Rusch et al., 2007).

We first report on analysis of individual metagenome sequences (average length ∼800 bp), then summarize results from metagenome assemblies. The G + C content (%) was determined for individual Sanger sequences (∼800 bp per read) from each of the six sites and classified taxonomically using “blastx” against the NCBI database (Figure 2). In addition, individual sequences were compared to available reference genomes using fragment-recruitment analysis (nucleotide) against more than 1400 reference genomes (tools developed during the Global Ocean Survey; Rusch et al., 2007) (Figure A1 in Appendix).

Classification of unassembled DNA sequence reads (Figure 2) showed that the acidic sites (DS_9 and OSP_14) were both dominated by *Hydrogenobaculum* populations with an average G + C content (%) of 34.7%. However, these two samples were considerably different with respect to the archaeal populations present. The elemental sulfur site (DS_9) contained novel populations within the Euryarchaeota and Thaumarchaeota, whereas the Fe-rich streamers (OSP_14) contained a *Metallosphaera yellowstonensis*-like population known to be an important Fe(II)-oxidizer in these habitats (Kozubal et al., 2011). Two circum-neutral sites (pH 6.5 and 7.8), which both contain high concentrations of dissolved sulfide, provided an excellent comparison to the lower pH (3–3.5) sulfidic habitats. A major shift from *Hydrogenobaculum*-like organisms at pH 3–3.4 (DS_9, OSP_14) to *Sulfurihydrogenibium*-like organisms at pH 6.5 and 7.8 (MHS_10 and CS_12) was observed across these environments. Nearly 90% of the unassembled Sanger sequence reads from MHS_10 were related to *Sulfurihydrogenibium* spp. (Figure 2). The G + C content distribution plots of sequences from MHS_10 and CS_12 show the importance of *Sulfurihydrogenibium*-like organisms with an average G + C value of 32.4%. A second major Aquificales population is evident in CS_12 with an average G + C content of 46.5%, and is closely related to the major Aquificales organisms present in OS_11 and BCH_13 (Figure 2). The relative abundance of Aquificales lineages observed using all random sequences was generally very similar to the distribution of 16S rRNA gene sequences PCR-amplified using universal bacterial primers (Figure A4 in Appendix).

The outflow channels of *Octopus* (OS_11) and *Bechler* (BCH_13) *Springs* exhibit similar pH values to CS_12 (pH 7.5–8), but do not contain significant concentrations of dissolved sulfide (below detection). The predominant Aquificales sequences in OS_11 and BCH_13 have nearly identical G + C content distributions (average G + C = 46.7%) and taxonomic assignment (*Thermocrinis*-like), and were also similar to the sub-dominant Aquificales population in CS_12 (Figure 2). Past work on the distribution of 16S rRNA genes in OS_11, BCH_13, and similar channels have also shown that these Aquificales are related to *Thermocrinis* spp. (Hugenholtz et al., 1998; Reysenbach et al., 2005; Hall et al., 2008). The only genome sequence available within this genus was *T. albus*, and this genome did not serve as an adequate reference for metagenome sequence from these sites (e.g., see fragment recruitment, Figure A1 in Appendix). Consequently, the YNP *Thermocrinis*-like populations are not well-represented by currently available reference genomes, and phylogenetic assignment of these population(s) in the G + C content frequency plot (Figure 2) was made at the family level (Aquificaceae). In contrast, the genome sequence of *Hydrogenobaculum* sp. Y04AAS1 is a reasonably good reference for populations present in DS_9 and OSP_14, while the *Sulfurihydrogenibium* spp. genomes (strain Y03AOP1 or *S. yellowstonense;* Reysenbach et al., 2009) serve as good references for metagenome sequence from MHS_10 and CS_12 (Figure A1 in Appendix). The recently released *Hydrogenobaculum* spp. genomes isolated from *Dragon Spring* (Romano et al., 2013) were not compared to DS_9 assemblies here, but these reference genomes are likely superior (relative to strain Y04AAS1) for direct comparison to *Hydrogenobaculum*-like sequence from DS_9.

### Phylogenetic analysis of metagenome sequence assemblies

**Figure 3:**
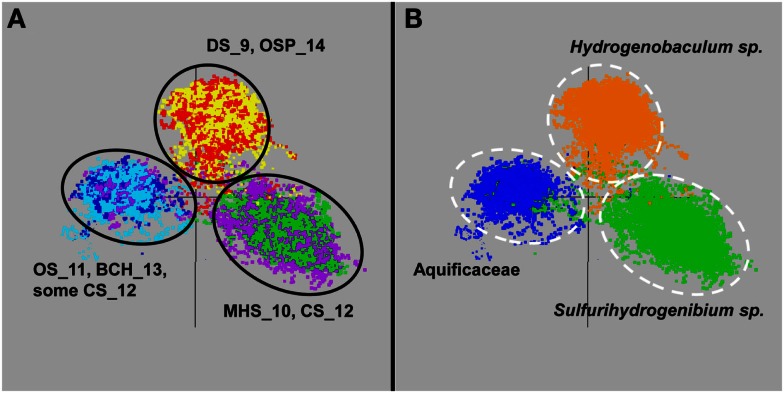
**Nucleotide word frequency PCA plots of metagenome assemblies from six Aquificales streamer communities in YNP**. **(A)** Data colored by site (black circles): DS_9 = yellow and OSP_14 = red; MHS_10 = green and CS_12 = violet; OS_11 = dark-blue and BCH_13 = light-blue. **(B)** Identical PCA orientation with phylogenetic analysis to the closest reference genomes (white-dashed circles): orange = *Hydrogenobaculum*, green = *Sulfurihydrogenibium*, dark-blue = Aquificaceae (no reference genomes adequately describe the similar *Thermocrinis*-like Aquificales phylotypes observed in OS_11 and BCH_13).

The *de novo* assemblies from each Aquificales community resulted in significant consensus sequence of indigenous populations present in these environments, regardless of whether the Celera (Rusch et al., 2007) or the PGA assembler (Zhao et al., 2008) was used. A similar number and distribution of contigs was generated from the two assemblies. In several cases, the amount of consensus sequence corresponding to the major Aquificales populations approached the expected size of reference Aquificales genomes (Reysenbach et al., 2009). Ordination of nucleotide word frequency analysis (NWF-PCA, or k-mer analysis) (Teeling et al., 2004) has been shown to be an excellent criterion for determining whether contigs generated during assembly may belong to the same species, and was used to evaluate all Aquificales contigs greater than 2000 bp (Figure 3). Similar Aquificales populations were observed in each of the replicate field sites: DS_9 (yellow) and OSP_14 (red); MHS_10 (green) and CS_12 (violet), and OS_11 (dark-blue) and BCH_13 (light-blue), respectively. Moreover, clear separation in Aquificales sequence was observed across sites with major geochemical differences (Figure 3A).

Phylogenetic analysis of these sequence clusters using the Automated Phylogenetic Identification System (APIS; Badger et al., 2006) in identical PCA orientation shows that the majority of Aquificales contigs from sites DS_9 and OSP_14 were most closely related to *Hydrogenobaculum* sp. Y04AAS1 (Figure 3B). Sequence clusters from MHS_10 consistently showed highest similarity to either the *Sulfurihydrogenibium* sp. Y03AOP1 or *S. yellowstonense* reference genomes. CS_12 contained Aquificales contigs most similar to the *Sulfurihydrogenibium* sp. genomes, as well as a separate population showing greater identity to the *Aquifex aeolicus* VF5 genome (i.e., Aquificaceae). In the absence of a reference strain with higher sequence identity, the *Aquifex aeolicus* VF5 genome served to represent this *Thermocrinis*-like population (see also Figure 2).

**Figure 4:**
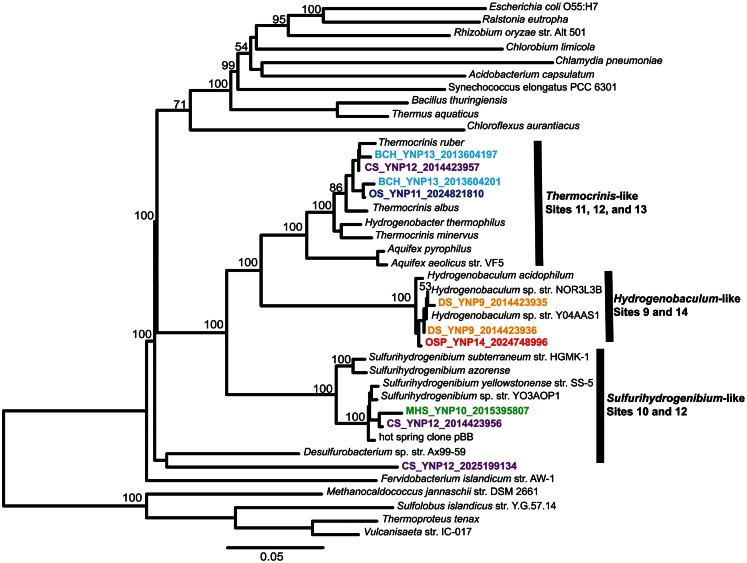
**Phylogenetic tree of Aquificales 16S rRNA gene sequences observed within assembled metagenome sequence from six YNP geothermal outflow channels [DS_9 (orange), OSP_14 (red), MHS_10 (green), CS_12 (violet), OS_11 (dark-blue), BCH_13 (light-blue) [neighbor-joining tree with 1000 bootstraps]**.

**Figure A2:**
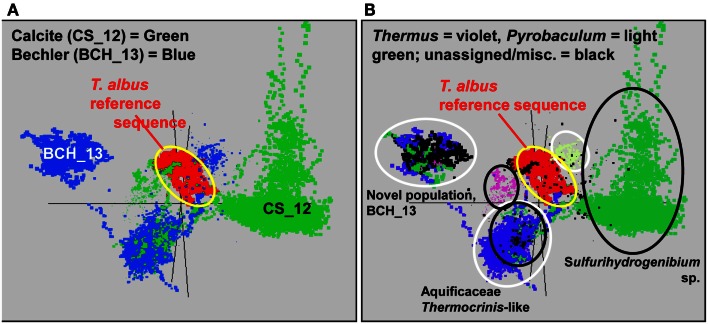
**Nucleotide word frequency PCA plots showing detailed analysis of Thermocrinis-like populations in Calcite and Bechler Springs compared to Thermocrinis albus reference sequence and other phyla present in these two communities**. **(A)** Sequence data colored by site (CS_12 and BCH_13), with *T. albus* reference sequence (red) added for comparison., and **(B)** Sequence data in identical orientation now analyzed phylogenetically to reveal specific assembled sequence corresponding to particular population types within the two different sites (green = *Sulfurihydrogenibium* sp., blue = Aquificaceae, violet = *Thermus* sp.; light-green = Thermoproteaceae; unassigned = black). White circles indicate major assemblies from BCH_13, and black circles indicate major assemblies in CS_12.

Contigs from the higher-pH (pH ∼8), non-sulfidic flow channels (OS_11 and BCH_13) cluster together and were also classified at the family level, Aquificaceae (Figure 3). As discussed above, the Aquificales populations within OS_11 and BCH_13 are actually related to *Thermocrinis* spp., as determined from phylogenetic analysis of 16S rRNA and other genes present within each of the metagenome assemblies (Figure 4). However, there are currently no adequate reference strains for comparison to these YNP populations. For example, the recently released *T. albus* genome is not a good reference for the YNP *Thermocrinis* populations from OS_11 or BCH_13, based on poor nucleotide identity and lack of similar gene content (Figure A2 in Appendix).

**Figure A3:**
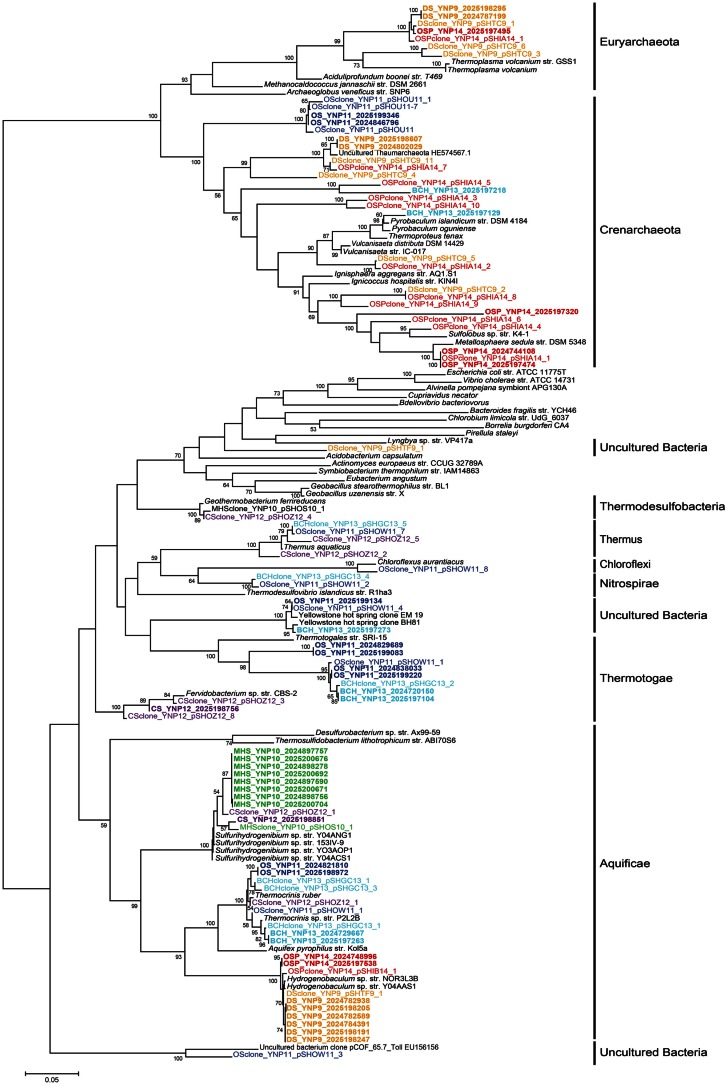
**Phylogenetic tree of all 16S rRNA genes observed in metagenome sequence (bold) combined with 16S rRNA genes amplified using PCR (up to 384 sequences per library) across six Aquificales streamer communities**. Entries are colored by site: DG_9 = orange; OSP_14 = red; MHS_10 = green; CS_12 = violet; OS_11 = dark-blue; BCH_13 = light-blue (core tree established using neighbor-joining methods with maximum-likelihood correction; metagenome entries added by parsimony).

**Figure A4:**
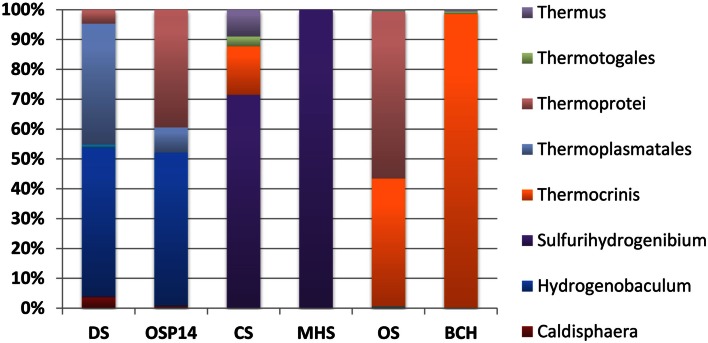
**Distribution of PCR-amplified 16S rRNA genes observed in ribosomal panels across Aquificales streamer habitats**. Universal archaeal and bacterial primers were used for each site and up to ∼384 clones were sequenced per primer set.

The composition of each community was also evaluated using phylogenetic analysis of 16S rRNA genes detected within the assembled sequence. Of 407 16S rRNA genes identified by the integrated metagenome data management and comparative analysis system (IMG/M, Markowitz et al., 2012) from all six sites, 64 were of sufficient length (>1100 bp) for phylogenetic analysis. Aquificales 16S rRNA gene sequences dominated the dataset (Figure 4) and grouped with *Hydrogenobaculum* (DS_9 and OSP_14), *Sulfurihydrogenibium* (MHS_10 and CS_12), or *Thermocrinis* spp. (OS_11, BCH_13, CS_12). Unclassified 16S rRNA gene sequences grouped with novel DNA sequences reported in previous studies (Reysenbach et al., 1994; Blank et al., 2002; Hall et al., 2008) and represent members of potentially deeply rooted candidate phyla (Hall et al., 2008). Finally, PCR-amplified bacterial and archaeal 16S rRNA gene clone libraries largely agreed with the diversity detected within the assembled metagenome sequence (Figures A3 and A4 in Appendix). Although archaeal primers failed to amplify from several samples, metagenome sequence contained significant levels of different archaea (Figure 2).

**Figure A5:**
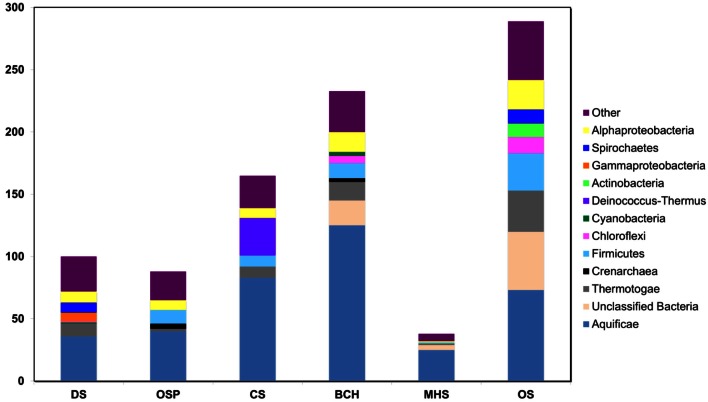
**Consensus phylogenetic classification (AMPHORA; Wu and Eisen, 2008) of assembled sequence using analysis of 31 housekeeping genes**. Although the Aquificales are the dominant members of each streamer sample, diverse and novel members of other bacterial and archaeal lineages are predicted to vary in abundance across sites.

To broaden our phylogenetic analysis, the community composition was also investigated by assigning phylotypes of 31 conserved protein-encoding marker genes using AMPHORA (Wu and Eisen, 2008). Of the 159,636 predicted genes among the six metagenomes, 994 were protein markers detected by AMPHORA and of sufficient length for phylogenetic analysis. At least 29 of the 31 housekeeping genes included in the AMPHORA database were detected in each of the six sites. The majority of marker genes for each site were assigned to members of the Aquificales, which reflects the greater amount of assembled Aquificales-like sequence across these sites, relative to other organisms (Figure A5 in Appendix). Significant amounts of assembled sequence corresponding to members of the Firmicutes, Alphaproteobacteria, Thermotogae, Bacteroidetes, Spirochetes, Proteobacteria, Actinobacteria, Deinococcus-Thermus, Acidobacteria, Gammaproteobacteria, Planctomycetes, Chlamydiae, Betaproteobacteria, Fusobacteria, and Epsilonproteobacteria, as well as unclassified *Bacteria* and *Archaea* was predicted using AMPHORA (Figure A5 in Appendix). A minor number of sequence matches to Chloroflexi, Chlorobi, and/or Cyanobacteria may reflect exogenous inputs from lower temperatures. The version of AMPHORA used here suggests greater diversity than indicated by other more direct phylogenetic analyses of either individual sequences, assembled contigs, or PCR-amplified 16S rRNA genes (e.g., Figures 2 and 3; Figures A2–A4 in Appendix), and is due in part to a limited number of appropriate reference genomes included in the AMPHORA database, especially from thermophiles and other deeply rooted, uncharacterized lineages. This prohibits accurate phylogenetic placement of a subset of these sequences. Consequently, the actual abundance of each population in these samples is better understood in terms of total sequence reads (i.e., Figure 2).

Population-level community richness can be inferred from the average abundance of single-copy genes detected within a specific population, assuming sufficient coverage (Table S1 in Supplementary Material). Given that a majority of the AMPHORA protein markers were detected for the Aquificales populations present in each site, the abundance of single-copy genes should provide a reasonable estimate of population-level heterogeneity. AMPHORA markers suggested that MHS_10 is dominated by a single *Sulfurihydrogenibium* sp., while CS_12 averaged five distinct *Sulfurihydrogenibium*-related copies of each single-copy gene (Table S1 in Supplementary Material). The acidic communities (DS_9 and OSP_14) may be comprised of three distinct *Hydrogenobaculum*-like populations, whereas OS_11 and BCH_13 contained at least eight or nine novel *Thermocrinis*-related populations. The number of Aquificales 16S rRNA genes identified in the metagenome assemblies (Figure 4) did not always agree with the number of Aquificales-related single-copy genes detected using AMPHORA. For example, 13 *Sulfurihydrogenibium*-like 16S rRNA genes were detected in MHS, yet the average abundance of single-copy genes suggest that this site is dominated by only one *Sulfurihydrogenibium*-like population. This discrepancy may be attributed to the fact that 16S rRNA genes do not often assemble with the same consistency as other housekeeping genes (Rusch et al., 2007), and/or to the possible presence of multiple rRNA operon copies (sequenced *Sulfurihydrogenibium* genomes have two to four annotated 16S rRNA gene copies). Although the sequencing depth obtained in the current study is not sufficient to accurately assess these communities at the ecotype level (Ward et al., 2006), it is clear that sequence variants of highly related Aquificales populations are evident within each site.

### Functional analysis of “Streamer” communities in YNP

**Figure 5:**
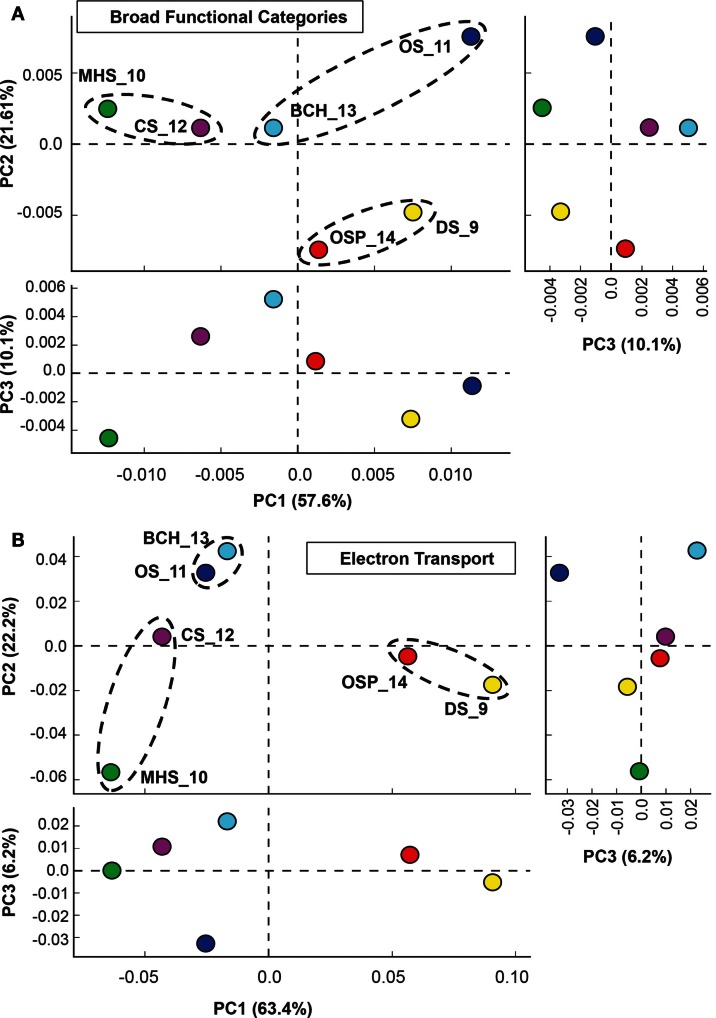
**Principal components analysis (PCA) of relative gene abundances across six Aquificales streamer communities**. **(A)** All TIGRFAMs grouped into functional categories, and **(B)** Only those TIGRFAMs associated with the role category “Electron Transport.” Site colors: DS_9 (yellow), OSP_14 (red), MHS_10 (green), CS_12 (violet), OS_11 (dark-blue), BCH_13 (light-blue). Circled sites represent “replicates” that contain one of three dominant Aquificales lineages (*Hydrogenobaculum, Sulfurihydrogenibium*, or *Thermocrinis*).

The functional gene content among the Aquificales “streamer” communities was compared using multivariate statistical analysis of protein family (TIGRFAM) abundances based on all predicted proteins from assembled metagenome sequence. To study different driving forces behind functional diversity, the analysis was performed using both the full set of all TIGRFAM protein families and a set of TIGRFAMS associated with electron transport (ET) functions. Both analyses reveal contributions from the predominant Aquificales lineage(s) across sites as well as the variable co-community members observed within each individual site (e.g., Figure 2). Site comparisons were made using PCA of all TIGRFAMS, which show strong support for the similarities between site pairs in acidic (DS_9, OSP_14) and circum-neutral sulfidic systems (MHS_10, CS_12) (Figure 5A). The first principal component explained over 57% of the total variation in protein family abundance among the different “streamer” communities and separated the two predominant families of Aquificales represented across these sites (Aquificaceae include both the *Hydrogenobaculum* sp. that dominate sites DS_9 and OSP_14, and the *Thermocrinis*-like populations in OS_11 and BCH_13). Also, these four sites all contained archaeal co-community members (e.g., ∼30% of sequence reads in OSP_14 and DS_9, ∼20% in OS_11, and ∼8% in BCH_13). Conversely, sites MHS_10 and CS_12 were dominated by *Sulfurihydrogenibium* populations in the family Hydrogenothermaceae. Despite the similar *Thermocrinis*-like populations present in OS_11 and BCH_13, these communities exhibited considerable functional differences, which ultimately tracked with differences in co-community members. Several of the phylogenetically distinct bacterial populations observed in OS_11 and BCH_13 (see Figure 2), which are not well characterized, contribute to unexpected functional differences between these communities. Factor 2, which accounted for ∼22% of variation in relative TIGRFAM abundance across these six sites, separated the low-pH *Hydrogenobaculum* sites from higher-pH sites.

Principal components analysis (PCA) using a subset of “ET” TIGRFAMS shows a slightly different pattern in site separation (Figure 5B) that is more sensitive to specific electron donors and acceptors (i.e., respiratory pathways), and more consistent with geochemical differences across sites (Table 1). Factor 1, which represented 63% of variation across sites, separated sites based on pH (low-pH sites DS_9 and OSP_14 versus other four sites), while Factor 2 correlated strongly with the presence or absence of sulfide, and separated the more oxic sites OS_11 and BCH_13 from sulfidic sites. The third principal component (∼6% of functional variation) emphasized differences in community composition between the two oxic sites (OS_11 and BCH_13), and did not contribute to overall functional differences in the more sulfidic systems (DS_9, OSP_14, MHS_10, CS_12).

**Figure 6:**
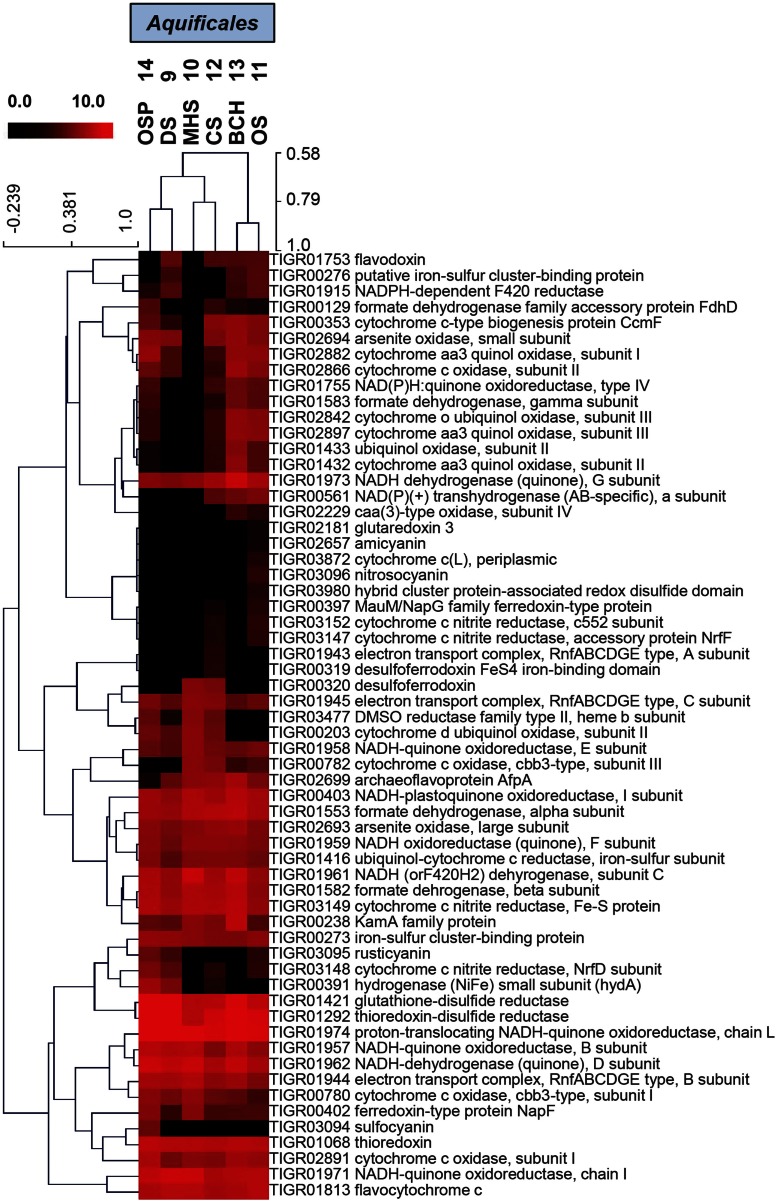
**Hierarchical cluster analysis of relative gene abundances in the TIGRFAM role category “Electron Transport” across six Aquificales streamer communities**. TIGRFAMs with low variation across the sites were removed before the clustering to retain ∼50 of the most variable families. Subunits of the protein complexes were only represented by one representative TIGRFAM family. Pearson correlation was used as the distance measure for average linkage agglomerative clustering. Sites cluster consistent with pH and the presence or absence of sulfide.

Hierarchical cluster analysis of the “ET” TIGRFAMs (Figure 6) show expected site pairing based on geochemical attributes identified in the study design (Inskeep et al., 2013), and can be used to identify specific protein families that explain the overall variation seen in PCA (Figure 5B). These TIGRFAMs included ET and terminal oxidase proteins specific to different respiratory pathways dependent on either sulfur, arsenite, hydrogen, and oxygen as well as other cytochromes, ferrodoxins, and flavoproteins (Figure 6; Table S2 in Supplementary Material). For example, while MHS_10 and CS_12 sites were both dominated by *Sulfurihydrogenibium* sp., the secondary *Thermocrinis* (family Aquificaceae*)* and *Thermus* populations in CS_12 contributed a significant number of additional cytochrome families that are not present in *Sulfurihydrogenibium* sp. Similarly, the higher complexity community at OS_11 contained several additional gene families compared to the lower complexity *Thermocrinis*-dominated community at BCH_13.

**Figure A6:**
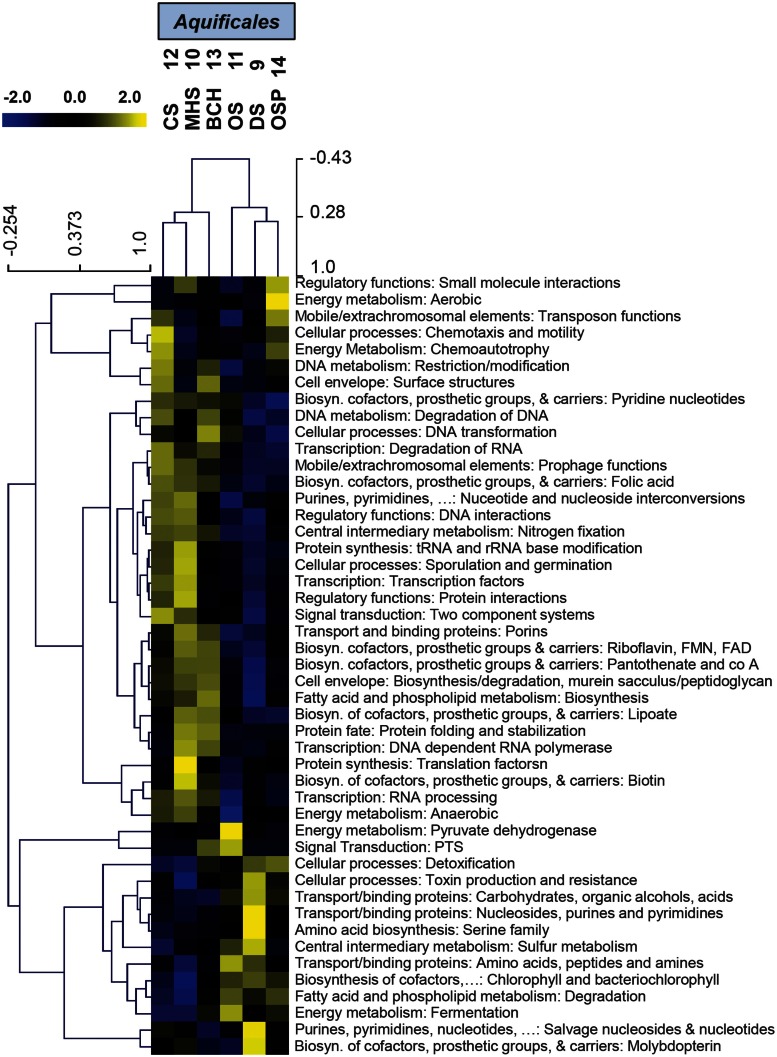
**Hierarchical cluster analysis of relative gene abundances across six Aquificales streamer communities using all TIGRFAMs grouped into functional categories**. Broad TIGRFAM categories include all cellular processes such as regulatory functions, energy metabolism, central C metabolism, mobile elements, transcription, cofactors and transporters. Data was standardized by functional category before clustering to avoid biasing analysis by a few categories with high gene abundance. Pearson correlation was used as the distance measure for average linkage agglomerative clustering.

Hierarchical cluster analysis using broader TIGRFAM profiles (Figure A6 in Appendix) supports the relative similarity of acidic sites (DS_9 and OSP_14) and circum-neutral sulfidic sites (MHS_10 and CS_12); however, the higher-pH non-sulfidic (i.e., oxic) communities (OS_11, BCH_13) did not form a separate group. Despite similar *Thermocrinis* populations in these two communities (e.g., see Figure 2), the overall functional profiles are quite different and this is consistent with the diverse and novel bacterial phylotypes observed in these sites (especially OS_11). Detailed analysis of specific functional categories across sites reveal differences in the relative abundance of genes coding for numerous cellular functions including nucleotide and DNA metabolism, regulatory functions, energy metabolism, central C metabolism, mobile elements, transcription, cofactors, and transporters (Figure A6 in Appendix).

**Figure A7:**
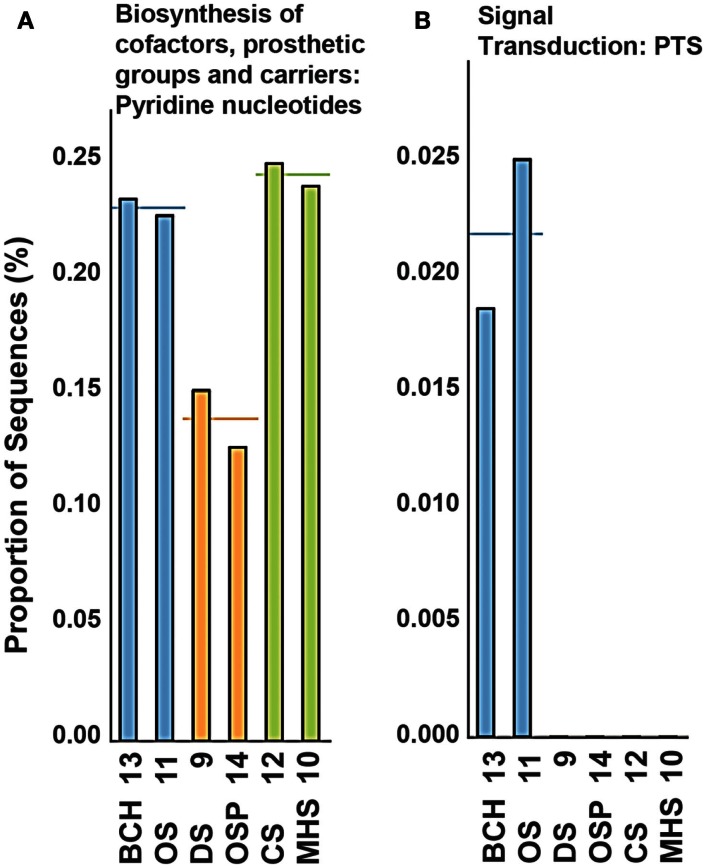
**Functional categories with the most significant differences in relative gene abundance among the six Aquificales streamer communities [(A) biosynthesis of cofactors, prosthetic groups and carriers and (B) signal transduction: PTS; *p*-values = 0.0047 and 0.0057, respectively]**. The relative proportion of sequences identified within each site are indicated (sites colored as before), and site pairs with similar geochemistry and Aquificales populations reveal replicate behavior in these specific TIGRFAMs.

**Figure A8:**
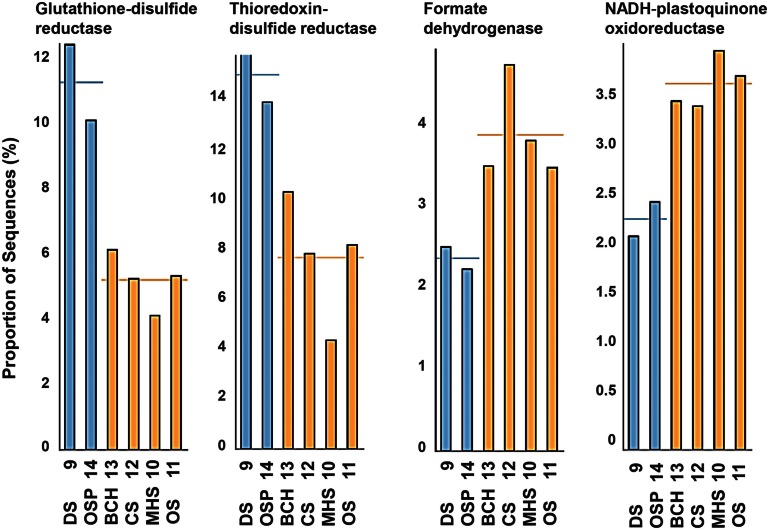
**Four most pH-dependent TIGRFAM families among six Aquificales streamer communities indicate a greater proportion of glutathione disulfide and thioredoxin-disulfide reductases in low-pH sites (blue bars) (sulfide present in both DS_9 and OSP_14) and a greater representation of formate dehydrogenases and NADH-plastoquinone oxidoreductases in higher-pH sites (orange bars) with (MHS_10, CS_12) or without (OS_11, BCH_13) sulfide**. The relative proportion of sequences to all electron transport-associated genes is shown for each streamer community.

The TIGRFAMs with the greatest statistical site separation (lowest *p*-value) include the pyridine nucleotide biosynthesis and PTS signal transduction categories (see Figure A7 in Appendix and Table S3 in Supplementary Material) for a complete list of TIGRFAM *p*-values). A coarse view of the importance of specific geochemical variables such as pH can be examined by looking at specific TIGRFAMs providing the highest statistical separation of sites based on selected variables. For example, the most pH-dependent TIGRFAMs include categories such as glutathione disulfide reductases, thioredoxin-disulfide reductases, formate dehydrogenases, and NADH-plastoquinone oxidoreductases (Figure A8 in Appendix).

### Functional differences among aquificales lineages in YNP

#### Fixation of carbon dioxide

**Figure 7:**
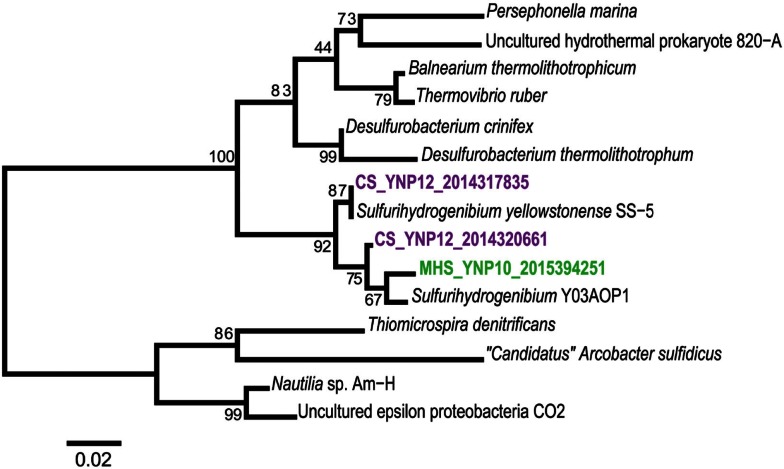
**Deduced protein tree of ATP citrate lyase (AclB), a key marker for CO_2_ fixation via the reverse-TCA cycle found in *Sulfurihydrogenibium* sp. in MHS_10 and CS_12 (neighbor-joining tree with 1000 bootstraps)**.

A detailed inventory of genes coding for carbon dioxide fixation and various oxidation/reduction pathways indicated major functional differences among the three primary Aquificales lineages observed in this study (Table 2). The reverse-TCA pathway is thought to be the earliest CO_2_ fixation process used by microorganisms (Wachtershauser, 1990; Hugler et al., 2005). Although members of the Aquificales have been shown to utilize this pathway (Aoshima et al., 2004; Ferrera et al., 2007), the enzymes associated with the key step (citrate cleavage) differ among families of the cultured Aquificales. For example, *Sulfurihydrogenibium* spp. (Family Hydrogenothermaceae) catalyze citrate cleavage using ATP citrate lyase, which includes a large and small subunit (AclA and AclB, respectively). Conversely, *Thermocrinis* spp. (Family Aquificaceae) catalyze citrate cleavage using two separate enzymes, citryl-CoA synthetase (Ccs) and citryl-CoA lyase (Ccl). We detected the *aclB* gene in both *Sulfurihydrogenibium* sites (MHS_10 and CS_12), but not in any sites containing strictly Aquificaceae (DS_9, OSP_14, OS_11 and BCH_13; Figure 7). Although CS_12 contained both *Thermocrinis* and *Sulfurihydrogenibium* populations, all *aclB* genes identified from this site grouped unambiguously with the *Sulfurihydrogenibium* sp. (Figure 7, bootstrap support = 92%).

**Table 2:** **Summary of key metabolic genes identified in metagenome sequence of the predominant Aquificales populations present in six different environmental systems covering a wide range in pH, dissolved sulfide, and dissolved oxygen**.

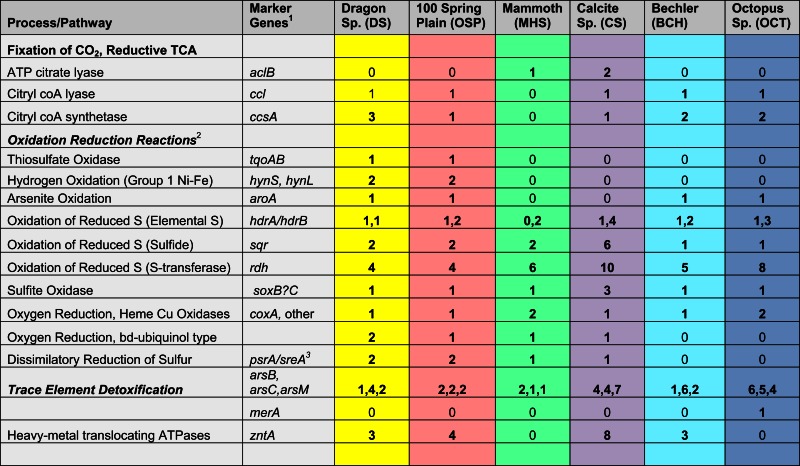

*^1^Marker genes code for proteins with high specificity for possible pathway*.

*^2^No genes were found for nitrification (amoA), denitrification (e.g. nirK, nirS, norB), methanogenesis or methanotrophy (e.g. mcrA), sulfate reduction (e.g. dsrAB), or arsenate reduction (e.g. arrAB) within the Aquificales populations represented in these six sites*.

*^3^Includes Mo-pterin proteins similar to sreA and arrA*.

**Figure 8:**
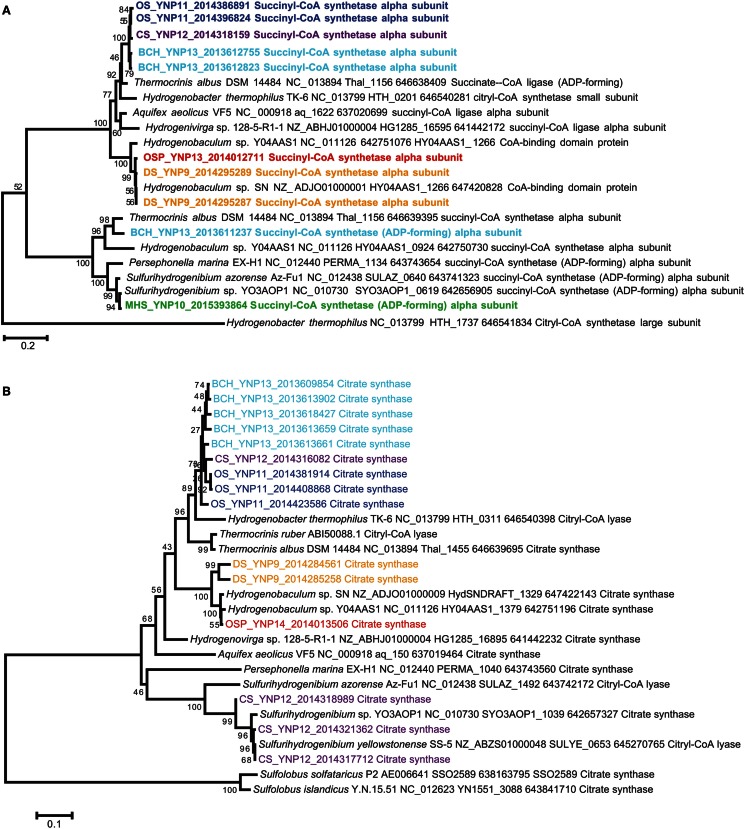
**Deduced protein tree of (A) citryl-coA synthetase (CcsA), and (B) citryl-CoA lyase (Ccl) showing metagenome entries from Aquificales sites (neighbor-joining tree with 1000 bootstraps)**.

Genes coding for citryl-CoA synthetase (*ccs*A) and citryl-CoA lyase (*ccl*) were found in both the *Hydrogenobaculum* (DS_9 and OSP_14) and *Thermocrinis*-like (OS_11, CS_12 and BCH_13) populations (Figures 8A,B). Therefore, these members of the Aquificaceae appear to fix CO_2_ using this alternative citrate cleavage mechanism. Deduced protein sequences (CcsA and Ccl) from OS_11, BCH_13, DS_9, and OSP_14 grouped in distinct clades consistent with the pH differences among sites, as well as the different genera observed within this family (i.e., DS and OSP versus OS and BCH). The *ccs*A gene copy detected in CS_12 grouped with similar entries in OS_11 and BCH_13, and was confirmed to come from the sub-dominant *Thermocrinis*-like population in this site. *Sulfurihydrogenibium*-like populations in MHS_10 and CS_12 lacked citryl-CoA synthetase (*ccs*A) genes, and instead contained one copy of a succinyl-CoA synthetase (Figure 8A). Differences in the gene neighborhood between the citryl-CoA and succinyl-CoA synthetase pathways of the Aquificaceae versus Hydrogenothermaceae suggest that the *Sulfurihydrogenibium* copy of succinyl-CoA synthetase from MHS is not involved in CO_2_ fixation. Annotation inconsistencies among these two fairly similar proteins (CcsA and succinyl-CoA synthetase) have made it difficult to make definitive assignments without visualizing the sequences in phylogenetic trees (Figure 8A) or other alignment tools, and these proteins are in fact thought to be related via gene duplication (Aoshima et al., 2004).

The r-TCA pathway has not actually been demonstrated in any cultured member of the *Hydrogenobaculum*, although field data on ^14^CO_2_ incorporation suggests that members of these communities fix CO_2_ at significant rates (Boyd et al., 2009). Genes found in DS_9 and OSP_14 are divergent relative to the *Thermocrinis* entries (Figures 8A,B), so it is unclear if these perform an identical function in both genera. However, other evidence that the r-TCA pathway is operative in the DS_9 and OSP_14 *Hydrogenobaculum*-like populations includes two enzymes required for the reductive pathway: 2-oxoglutarate:ferredoxin oxidoreductase and fumarate reductase. Both genes are present in the *Hydrogenobaculum* sp. Y04AAS1 genome as well as the DS_9 and OSP_14 metagenomes.

##### Oxidation of H_2_, reduced sulfur, and arsenite

Other functional differences among the major Aquificales lineages include possible explanations for the specialization of these populations to specific geochemical environments. For example, *Hydrogenobaculum* populations from DS_9 and OSP_14 contain Group I Ni-Fe hydrogenases, but these genes are notably absent from the *Sulfurihydrogenibium* (MHS_10 and CS_12) and the *Thermocrinis* populations (OS_11 and BCH_13) (Table 2). The potential for H_2_ to serve as an electron donor for metabolism appears limited to the acidic sites where concentrations of aqueous H_2_ have been measured in the 50–100 nM range (Inskeep et al., 2005; Spear et al., 2005), while other sites contain lower H_2_ (Table 1). The *Hydrogenobaculum*-like populations were also the only Aquificales to contain genes coding for thiosulfate oxidase (*tqoAB*), often implicated in oxidation of thiosulfate in the order Sulfolobales (Friedrich et al., 2005). However, it is known that thiosulfate concentrations are considerably higher in circum-neutral sulfidic sites, due to the greater stability of thiosulfate at intermediate pH (Xu et al., 1998; Nordstrom et al., 2005). The *Sulfurihydrogenibium-*like organisms may process thiosulfate through an abundance of rhodanese domain proteins known to be involved in sulfur-transferase reactions as well as SoxBC complexes (Friedrich et al., 2005). Moreover, all Aquificales lineages from each site contained either one or more copies of a highly conserved and syntenous gene complex thought to be important in the oxidation of reduced sulfur (i.e., sulfide and elemental S), and includes several hetero-disulfide reductases as well as other Fe-S proteins (*rhd*, *tusA*, *dsrE*, *hdrC*, *hdrB*, *hdrA*, *orf2*, *hdrC*, *hdrB)* (Ghosh and Dam, 2009). Each of the three lineages also contained *sqr* (sulfide:quinone reductase) genes (Table 2), which have been shown to code for proteins involved in the oxidation of dissolved sulfide to S^0^ or polysulfide chains, followed by electron transfer to the quinone pool through a flavin adenine dinucleotide (FAD) cofactor (Cherney et al., 2010). Even the *Thermocrinis* populations from OS_11 and BCH_13 exhibited potential for the oxidation of sulfide and elemental S, although it is unlikely that sufficient sulfide exists in these geothermal channels to support the growth of active “streamer” communities. The habitat range of *Thermocrinis*-like organisms in YNP includes high-pH (7–9) sulfidic channels (Inskeep et al., 2005; Hall et al., 2008; Planer-Friedrich et al., 2009) and thus may explain the presence of these genes in *Thermocrinis* assemblies. The HDR gene complexes appear highly conserved across numerous Aquificales and Sulfolobales, as well as acidophilic bacteria such as *Acidithiobacillus ferrooxidans* (Ghosh and Dam, 2009; Inskeep et al., 2013).

The oxidation of arsenite to arsenate is highly exergonic (ranging from 50 to 60 kJ/mol electron) in these geothermal systems (Inskeep et al., 2005) and has been shown to serve as a sole electron donor in several unrelated bacteria (D’Imperio et al., 2007; Santini et al., 2007). Consequently, it is interesting that both the *Hydrogenobaculum* and *Thermocrinis*-like organisms from sites DS_9, OSP_14, OS_11, and BCH_13 contain full copies of the arsenite oxidase Mo-pterin subunit I (*aio*A, also abbreviated *aro*A, *aso*A, *aox*B in prior work). *Thermocrinis* and *Hydrogenobaculum* spp. oxidize considerable amounts of arsenite in acidic to circum-neutral springs in YNP (Macur et al., 2004; Inskeep et al., 2005; Hamamura et al., 2009), corresponding to the oxygenation of geothermal outflow channels and correlation with *aio*A expression in these same habitats (Clingenpeel et al., 2009; Hamamura et al., 2009). It is possible that members of the Aquificales gain energy from the oxidation of arsenite *in situ*. However, this has not been established in culture (Donahoe-Christiansen et al., 2004). All Aquificales populations from the current study also contained evidence for arsenic detoxification, including the potential to reduce arsenate via ArsC and extrude arsenite via an efflux pump (AsrB), as well as to methylate arsenite via methyl transferases (ArsM) (Bentley and Chasteen, 2002; Mukhopadhyay et al., 2002).

##### Respiratory processes

**Figure 9:**
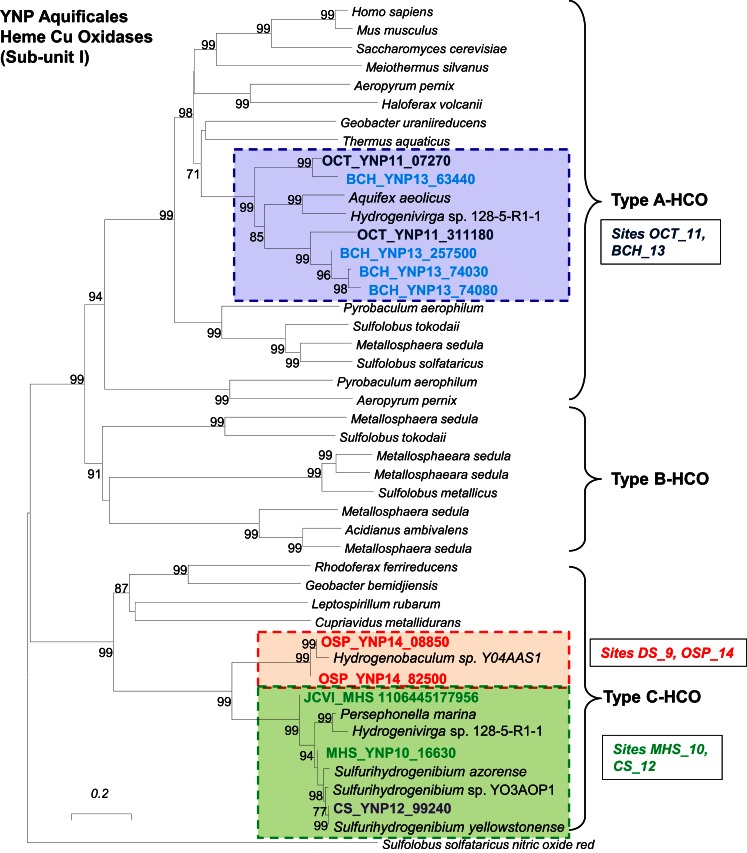
**Protein tree of heme-copper oxidases (subunit I of terminal oxidase complex)**. Metagenome entries are highlighted by site, and labels correspond to the major types of heme-copper oxidases observed in different Aquificales “streamer” communities. Similar heme-copper oxidases are found in sites OS_11 and BCH_13, and these are significantly different than the terminal oxidases found in *Hydrogenobaculum* and or *Sulfurihydrogenibium* from sites DS_9, OSP_14, MHS_10 and CS_12 (neighbor-joining tree constructed using nitric oxide reductase (NorB) as the out group; 1000 bootstraps).

The presence of terminal oxidase complexes in each of the Aquificales populations suggests that these organisms all respire oxygen. However, the distribution of different types of subunit I heme Cu oxidases (HCOs) across the Aquificales lineages (Figure 9) is not consistent with the distribution of CO_2_ fixation genes. This observation invokes a different evolutionary history of carbon dioxide fixation versus aerobic respiration among these Aquificales lineages. The *Sulfurihydrogenibium* and *Hydrogenobaculum-*like organisms contain similar Type C-Cbb3 HCOs (Garcia-Horsman et al., 1994) despite the phylogenetic distance between these organisms (i.e., different families). Conversely, the *Thermocrinis* spp. from OS_11 and BCH_13 contained multiple copies (at least two distinct copies per site) of Type A HCOs (Pereira et al., 2001), which are phylogenetically related to HCOs from the majority of aerobic bacteria.

*Hydrogenobaculum* (DS_9, OSP_14) and *Sulfurihydrogenibium*-like (MHS_10, CS_12) organisms were the dominant Aquificales in sites containing high levels of dissolved sulfide (low dissolved O_2_). Consequently, the Cbb3 cytochromes associated with these populations likely bind O_2_ with greater efficiency compared to Type A HCOs of the *Thermocrinis*-like populations, and is consistent with known properties of these proteins (Garcia-Horsman et al., 1994). The Cbb3-type heme-copper oxidases are found only in several groups of bacteria, especially the Aquificales and Proteobacteria (312 of 365 sequences; Sousa et al., 2011), and appear to represent the evolution of a separate respiratory complex in low O_2_ environments (Garcia-Horsman et al., 1994). In addition, the *Hydrogenobaculum* and *Sulfurihydrogenibium*-like organisms have genes coding for a *bd*-ubiquinol oxidase (*cydAB*), also thought to function under lower O_2_ concentrations (Jünemann, 1997). The *Thermocrinis*-like organisms in OS_11 and BCH_13 show no evidence of the *bd*-ubiquinol oxidases, consistent with the higher oxygen levels and low sulfide in these sites.

A detailed survey of other respiratory processes (dissimilatory reduction) suggests that the reduction of elemental sulfur and/or polysulfide is important in some members of the Aquificales. Although some sequenced Aquificales isolates contain a nitric oxide reductase (NorB) (one of the steps required for complete denitrification from nitrate to N_2_), *nar*G, *nor*B, *nir*K, or *nir*S like sequences were not found in the YNP Aquificales. Moreover, no evidence was found for dissimilatory reduction of sulfate or sulfite (*dsr*AB), arsenate (*arr*AB), or CO_2_ (*mcr*A, methanogenesis) in any of the Aquificales lineages. However, the organisms found in sulfidic channels (i.e., *Hydrogenobaculum* and *Sulfurihydrogenibium*) all contain sulfur reductases (*sre*A) or polysulfide reductase (*psr*A), as well as tetrathionate reductases (*ttr*A, another DMSO-Mo-pterin). Consequently, the Aquificales lineages that inhabit sulfidic channels under low O_2_ tensions may require electron shuttling to reduced sulfur instead of, or in addition to, O_2_. Genes for sulfur reduction were notably absent in the *Thermocrinis-*like populations of OS_11 and BCH_13, which correlates with the lack of elemental sulfur in these springs.

## Discussion

Phylogenetic and functional analysis of metagenome sequence from three major types of high-velocity, filamentous “streamer” communities revealed three lineages of Aquificales, whose metabolic potential correlated primarily with pH and sulfide and/or elemental sulfur. Sites with low-pH (pH 3–3.5) and high-sulfide contained *Hydrogenobaculum* spp., whereas higher-pH sites were dominated by either *Sulfurihydrogenibium* spp. (high-sulfide) or *Thermocrinis*-like (low sulfide) populations. *Calcite Springs* (CS_12) also hosted a minority *Thermocrinis*-like population and was the only site here to contain two major Aquificales genera. This is consistent with previous 16S rRNA gene diversity surveys that have generally found only minor overlap in the distribution of different Aquificales across YNP geothermal environments (Reysenbach et al., 2005; Hall et al., 2008; Hamamura et al., 2009). *Thermocrinis* organisms have also been observed in sulfidic channels at higher-pH, near 9 (Planer-Friedrich et al., 2009).

Metagenome sequence assemblies for each of the three major Aquificales lineages resulted in total scaffold sizes that approach full genomes, and which represent “consensus sequence” or “pan-genomes” of these populations (Medini et al., 2005). Sequence variability of highly related populations within a field site may contribute to incomplete assembly, and “closure” of these *de novo* assemblies would require considerable manual effort, as well as additional sequencing to close gaps. Sequence heterogeneity within individual Aquificales populations was observed using AMPHORA to detect the number of single-copy genes. Although the streamer community from MHS_10 was dominated by what appears to be a fairly homogeneous population type of *Sulfurihydrogenibium* sp., other sites exhibited greater variability within the primary Aquificales population. For example, the *Thermocrinis*-like populations from *Octopus*, *Bechler*, and/or *Calcite Springs* all revealed higher numbers of numerous single-copy genes, which suggests greater heterogeneity of these populations *in situ*. Additional sequence coverage of these populations may result in less single-copy gene variability, and future efforts will be necessary to clarify sources of this variability. Importantly, the sequence assemblies generated in this study provide a foundation for future efforts to determine the types and rates of genetic change in these same sites.

In addition to the abundant Aquificales populations, *de novo* assemblies were also obtained for several novel bacterial and archaeal lineages, although at lower coverage. These co-community members provide an interesting comparative study in their own right given that each Aquificales community exhibited a different assemblage of interacting community members. For example, the acidic sites contained different archaeal populations representing specialization on Fe(II) (e.g., *Metallosphaera*-like) versus reduced sulfur (Thaumarchaeota- and Thermoplasmatales-like), while the higher-pH (pH ∼8) sites contained several novel bacterial lineages, and a population of Thermoproteaceae (G + C ∼ 60%) in OS_11 and BCH_13. Some of the novel bacterial populations present in OS_11 and BCH_13 were related to unclassified 16S rRNA gene sequences described previously (Reysenbach et al., 1994; Blank et al., 2002; Hall et al., 2008). Metagenomics provides a promising opportunity to gain insight into the metabolic potential of these novel populations, although additional sequencing will be required to bring the coverage up to levels suitable for building reasonable *de novo* assemblies (e.g., >2× coverage).

Global protein (TIGRFAM) analysis coupled with PCA and hierarchical clustering of all Aquificales “streamer” communities demonstrated important linkages among geochemistry, the presence of distinct phylotypes, and their metabolic genes. Clustering of TIGRFAMs specific to ET demonstrated specific linkages between individual phylotypes and respiratory pathways that are consistent with the strong influence of geochemistry on community structure (especially pH and sulfide). Perhaps one of the more interesting findings in the study is the degree to which co-community members varied across these high-velocity stream-channel habitats. Even site pairs containing the same Aquificales phylotype contained considerably different interacting populations. Factors controlling the variation in community structure in different sites containing the same Aquificales phylotype can also be shown to track with geochemical conditions. Such is the case when comparing the hypoxic elemental sulfur habitats (DS_9) to the more oxic Fe(III)-oxide (OSP_14) streamer communities, samples that both contained a very similar *Hydrogenobaculum* population. The archaeal co-community members in DS_9 are likely anaerobic (or microaerophilic) populations compared to the aerobic Fe(II)-oxidizing Sulfolobales in OSP_14.

Detailed analysis of functional genes present in the three Aquificales lineages revealed several examples of divergence that were likely driven by environmental selection and lateral transfer events. These events have resulted in inconsistent patterns in phylogeny between highly conserved genes and those coding for various functional processes. Characteristics of the r-TCA cycle make it a good candidate for analysis of early steps important in the evolution of autotrophy. The pathway’s auto-catalytic nature and role in central C metabolism may reflect its importance in the evolution of associated anabolic and oxidative pathways (Hugler et al., 2005). The key ATP-dependent step of acetyl CoA synthesis is catalyzed by different proteins in the Hydrogenothermaceae versus Aquificaceae (AclB versus CcsA and Ccl, respectively; Aoshima et al., 2004), suggesting different evolutionary histories of these lineages with respect to CO_2_ fixation. The only Aquificales in this study to contain *acl*B were the *Sulfurihydrogenibium*-like organisms (Family Hydrogenothermaceae) present in MHS and CS (Figure 7). The *Hydrogenobaculum* and *Thermocrinis* populations (Family Aquificaceae) in the other sites contain *ccs*A and *ccl* genes rather than *acl*B (Figures 8A,B). The r-TCA pathway catalyzed by Ccs and Ccl is believed to be the ancestral pathway among the Aquificales (Hugler et al., 2005), however, our understanding of this pathway is not complete as evinced by inconsistent annotation of both the citryl-CoA synthetase (*ccs*A) and the citryl-CoA lyase (*ccl*).

Although the Aquificales occupy diverse geochemical habitats, they generally flourish in zones of shallow, high-velocity, turbulent spring water where dynamic mixing occurs to create disequilibria between hypoxic and oxic conditions. The exchange of atmospheric oxygen and subsequent reaction with dissolved sulfide present in thermal waters is one of the more important geochemical processes occurring within sulfidic geothermal channels (Inskeep et al., 2005; Nordstrom et al., 2005). The disequilibrium between oxygen and sulfide establishes conditions suitable for microaerophiles growing on sulfide, elemental sulfur or thiosulfate (Reysenbach et al., 2009). Metagenome sequence of the YNP Aquificales clearly indicates the potential for the oxidation of reduced sulfur species using a variety of S-oxidation pathways coupled with Type C-Cbb3 or *bd*-ubiquinol terminal oxidase complexes, especially in the *Hydrogenobaculum* and *Sulfurihydrogenibium-*like organisms detected in sulfidic systems. The *Thermocrinis* organisms present in OS_11 and BCH_13 contain Type A-HCOs (Figure 9), indicating their functional divergence from the other Aquificales genera with respect to oxygen. There is evidence that some Aquificales have copies of both types of HCOs as is noted for *Hydrogenivirga* spp. Consequently, it is possible that subsequent evolution in specific habitat types separated lineages containing only the Type C or the Type A HCO. Interestingly, the *Hydrogenobaculum* and *Sulfurihydrogenibium* populations both contain the cbb3-Type C HCOs even though they are members of different families. The *Hydrogenobaculum* and *Thermocrinis* organisms are from the same family, but do not share the same respiratory complexes, although they do share similarity in r-TCA proteins important in CO_2_ fixation. The major difference in metabolic processing of CO_2_ and O_2_ between these lineages provides an excellent opportunity for relating their evolutionary histories to paleobiological events and the timing of radiation relative to the “Great Oxidation Event” (Canfield, 2005; Anbar et al., 2007; Konhauser, 2009).

The two higher-pH (pH ∼8) non-sulfidic sites (OS_11, BCH_13) contained a similar *Thermocrinis*-like Aquificales, however, the microbial community structure was considerably different and the OS_11 streamer community contained at least three additional novel bacterial assemblies compared to BCH_13. The inorganic constituents of these two springs were reasonably similar and they both supported a similar *Thermocrinis*-like population. Clearly, additional geochemical and or geophysical factors, not considered in the current study, contribute to these differences in community structure across apparently similar sites. Variations in dissolved and/or particulate organic carbon across sites may play an important role in modifying community composition. However, the organic compounds contributing to the measured total dissolved organic carbon (DOC), as well as solid-phases of C, have not been characterized. Although the concentration of DOC was higher in OS_11 than BCH_13, this association is not supported with any detailed linkages among specific organic compounds and microbial diversity at the current time. Further characterization of organic constituents present in geothermal systems will be necessary to determine if variations in organic solutes contained in geothermal source waters also influence community structure and function across different habitat types. Moreover, additional sites are necessary to understand possible differences in *Thermocrinis*-like populations present in high-sulfide environments (e.g., CS_12) compared to those in low sulfide systems (e.g., OCT_11, BCH_13).

## Materials and Methods

### Site selection, sample collection, and processing

Six Aquificales “streamer” communities (Figure 1) were sampled from high-velocity, in-channel habitats during 2007–2008, and were chosen to replicate at least three major lineages of Aquificales known to exist in YNP across a pH range from 3 to 9 (i.e., *Hydrogenobaculum*, *Sulfurihydrogenibium*, and *Thermocrinis* spp.). The research sites chosen for study have been the subject of significant prior characterization and include: *Dragon Spring* (DS_9), *One Hundred Springs Plain* (OSP_14), *Mammoth Hot Spring* (MHS_10), *Calcite Springs* (CS_12), *Octopus Spring* (OS_11), and *Bechler Springs* (BCH_13) (e.g., Fouke et al., 2003; Inskeep and McDermott, 2005; Inskeep et al., 2005; Reysenbach et al., 2005; Fouke, 2011). Each microbial community and associated solid phase was sampled aseptically, stored in 50 mL sterile Falcon tubes on dry ice, and transported to a −80°C freezer (MSU) until DNA extraction.

Parallel samples of the bulk aqueous phase (<0.2 μm) associated with the microbial community were obtained simultaneously and analyzed using a combination of field and laboratory methods. As described in more detail in other reports (Inskeep et al., 2004; Macur et al., 2004; Hall et al., 2008), pH, temperature, and other redox sensitive species (Fe^2+^/Fe^3+^; As^III^/As^V^; total dissolved sulfide; dissolved O_2_) were determined using field methods. Total dissolved ions were determined using inductively coupled plasma (ICP) spectrometry and ion chromatography (for all major cations, anions, and trace elements). Dissolved gases (CO_2_, H_2_, CH_4_) (all sites but BCH) were determined using closed head-space gas chromatography (Inskeep et al., 2005) of sealed serum-bottle samples obtained in the field. A subset of these sites have been sampled many times with excellent replication (Langner et al., 2001; Inskeep et al., 2005, 2010; Reysenbach et al., 2005; Fouke, 2011). The location and primary physicochemical characteristics obtained during sampling are provided here (Table 1), and additional geochemical data are provided as supplemental information (Table S2 in Supplementary Material, Inskeep et al., 2013).

### DNA extraction and library construction

DNA was extracted from all samples using a standardized protocol (Inskeep et al., 2013) to minimize variation in composition across sample type due to extraction method or technician. Our main emphasis was to obtain representative, unbiased community DNA as template for construction of small insert libraries. Briefly, approximately 3 g wet samples were extracted in 1 ml of Buffer A (200 mM Tris, pH 8; 50 mM EDTA; 200 mM NaCl; 2 mM sodium citrate; 10 mM CaCl_2_) with lysozyme (1 mg/ml final concentration) for 1.5 h at 37°C. Proteinase K (final concentration 1mg/ml) and SDS [final concentration 0.3% (w/v)] were added and incubated for 0.5 h at 37°C. This first lysate was removed, and the samples were re-extracted using bead-beating protocols. The two lysates were then combined and extracted with phenol-chloroform, and the resulting DNA was re-precipitated in ethanol, treated with RNAase and quantified by gel electrophoresis and staining. Small insert (puc13) libraries were constructed and transformed, then sequenced using Sanger sequencing to generate approximately 40 Mbp per site (∼800 bp reads), with the exception of MHS_10 that only received ∼20 Mbp of Sanger sequence, due in part to the simplicity of the community and the fact that MHS_10 also received a half-plate of 454 pyrosequencing. For consistency, this manuscript focuses on the Sanger data across each of the six sites; assemblies using the pyro-sequence data for MHS_10 did not result in improved contig size or increases in total assembled data, although it did improve the coverage of this phylotype to >50×. Full-length 16S rRNA genes were also PCR-amplified and cloned from the DNA of each site using universal primers specific for *Bacteria* and *Archaea*, and one 384-well plate was sequenced from each successful library. Unique PCR-amplified 16S rRNA gene sequences (<97% DNA similarity) were chimera-checked using Bellerophon (Huber et al., 2004).

### Metagenome sequence analysis

Unassembled metagenomic sequence reads were plotted as a function of %G + C content and taxonomic assignment based on best “blastx” hits using MEGAN (Huson et al., 2007). Only a handful of microbial genomes currently serve as appropriate references for the indigenous organisms within these chemotrophic communities, consequently, many of the taxonomic assignments were given at family or domain level. Genome-level analysis of metagenome data was performed using fragment recruitment of unassembled sequence reads to reference microbial genomes (Rusch et al., 2007). At the time of writing, this database contained reference microbial genomes for ∼1500 bacteria and 100 archaea.

Random shotgun DNA sequence (∼40–50 Mb Sanger per site) was assembled using both the Celera (Version 4.0, Rusch et al., 2007) and PGA (Zhao et al., 2008) assemblers as described previously in Inskeep et al. (2013). Briefly, the analyses presented here was based on the Celera assemblies of our metagenomic data built using the following parameters: doOverlapTrimming = 0, doFragmentCorrection = 0, globalErrorRate = 12, utgErrorRate = 150, utgBubblePopping = 1, and useBogUnitig = 0. For PGA assemblies (Zhao et al., 2008), the following parameters were employed: OverlapLen = 30; Percent = 0.75; Clearance = 30; ClipIdn = 77; ClipQual = 10; CutoffScore = 400; EndOverhang = 800; InOverhang = 500; MinCovRep = 50; MinLinks = 2; MinSat = 3; NumIter = 50; PenalizeN = 1; QualOverLim = 400; QualScoreCutoff = 200; QualSumLim = 3500; SimDiFac = 30; Verbosity = 1. Assemblies obtained from Celera and PGA were gene-called and annotated using the Department of Energy-Joint Genome Institute IMG/M pipeline (Markowitz et al., 2012). All annotated metagenome sequence assemblies (Celera/PGA) discussed in the current manuscript are available through the DOE-JGI IMG/M (Markowitz et al., 2012) website (http://img.jgi.doe.gov/m) under IMG taxon OID numbers as follows: YNPSite09 (2022920010/2014031004), Site14 (2022920007/2013954001), Site10 (2022920015/2015391001), Site12 (2022920011/2014031005), Site11 (2022920012/2014031007), and Site13 (2022920006/2013515002).

Assembled metagenome sequence (e.g., contigs and scaffolds > 2 kb) was analyzed using three dimensional PCA scatterplots of nucleotide word frequencies (Teeling et al., 2004; Inskeep et al., 2010) to evaluate consensus assembled sequence of dominant phylotypes (e.g., Figure 3A). The sequence clusters were also viewed (Figure 3B) with a simultaneous blast-based taxonomic classification (Rusch et al., 2007) or the Automated Phylogenetic Inference System (APIS; Badger et al., 2006). Briefly, APIS is a system for automatic creation and summarizing of phylogenetic trees for each protein encoded by a genome or metagenomic dataset.

### Phylogenetic marker and single-copy genes

The community composition of each site was also investigated by phylogenetic analysis of 16S rRNA genes detected among the six assembled metagenomes in IMG/M. Of the 407 sequences annotated as 16S rRNA genes, 64 were of sufficient length (>1100 bp) for robust phylogenetic analysis. Smaller fragments were not included in the alignments or phylogenetic tree to maximize robust phylogenetic placement of the primary community members. 16S rRNA gene sequences were aligned in Green genes (DeSantis et al., 2006), imported into the ARB program (Ludwig et al., 2004), and manually adjusted according to conserved regions of the gene and the established secondary structure to ensure that only homologous regions were compared. Initial phylogenetic analysis was performed in PAUP* (version 40.b10; Sinauer Associates, Sunderland, MA, USA) and tree topology was explored using parsimony, neighbor-joining, and maximum-likelihood analyses. Final 16S rRNA gene trees (Figure 4; Figure A3 in Appendix) were created by neighbor-joining analysis with a maximum-likelihood correction. For the Aquificales-specific tree (Figure 4), a heuristic search was performed with tree bisection-reconnection (TBR) branch swapping in PAUP*. Transition/transversion ratio and nucleotide frequencies were estimated according to the F84 model and bootstrap values were determined from 1000 re-samplings of the dataset. 16S rRNA genes from metagenome assemblies and unique (<97% DNA similarity) PCR-amplified 16S rRNA genes from the original sample DNA (up to 384 sequences per library were amplified using universal bacterial and archaeal primers and chimera-checked using Bellerophon; Huber et al., 2004) were added to a neighbor-joining 16S rRNA gene tree (Figure A3 in Appendix) using the parsimony tool in ARB.

Community composition was also investigated by identifying the phylogeny of 31 conserved protein-encoding marker genes using AMPHORA (Wu and Eisen, 2008). Assembled contigs and singleton reads from the six sites were analyzed as described for the Sargasso Sea dataset in Wu and Eisen (2008). Phylotypes were identified from the first internal node (n1) whose bootstrap support exceeded 70%. Phylogenetic classifications that had less than 70% bootstrap support were resolved further by “blastx identity.” For example, because there are few archaeal representatives included in the AMPHORA database, significant bootstrap support was not often detected for archaeal query sequences. Similarly, deeply divergent sequences were poorly supported and were unidentified in AMPHORA, thus requiring further investigation using “blastx.” Aquificales single-copy genes that were shared among all datasets (*n* = 911 total distributed among 29 protein markers) were identified and population richness was determined as the average number of single-copy genes present in each dataset (Table S1 in Supplementary Material).

### TIGRFAM analysis

Assembled metagenome sequence from each of the “streamer” communities was annotated as described in Inskeep et al. (2010, 2013) and predicted proteins from the scaffolds were assigned TIGRFAM protein families (Selengut et al., 2007) using HMMER 3 (Eddy, 2011) with E-value cutoff of 1e-6. PCA and statistical analysis of site group differences was performed using the STAMP v2.0 software (Parks and Beiko, 2010). Briefly, the White’s non-parametric *T*-test and ANOVA tests were used to test for differences between two site groups and multiple site groups, respectively. Two-way clustering was done using row-standardized (across sites) average TIGRFAM category abundance data using the Euclidean distance metric and complete-linkage hierarchical clustering in MeV 4.8 (Saeed et al., 2003) software.

### Functional comparisons among aquificales lineages in YNP

The assembled metagenome sequence data was screened for specific functional genes corresponding to known and or putative pathways involved in biosynthesis and energy transfer. Specifically, we were interested in assessing metabolic potential for chemolithoautotrophy (CO_2_ fixation and electron transfer genes) in high-temperature geothermal systems. Query DNA sequences known to code for proteins important in the oxidation of reduced chemical constituents or the reduction of a terminal acceptor were used to search the assembled metagenome sequence data using “blastx” routines (full list of gene sequences and accession numbers given Table S3 in Supplementary Material, in Appendix, Inskeep et al., 2013). IMG/M was used as an additional method for identifying CO_2_ fixation and other functional genes, and for gene neighborhood analysis. Metagenome sequences exhibiting homology (*E*-values < 10^−10^) to query sequences were then carefully assessed by manually examining amino acid sequence alignments (for fragments of sufficient length relative to query sequence) and subsequent phylogenetic analysis of deduced protein sequences against known relatives. False positives were eliminated by this screening process and included (i) sequences matching the correct protein family of the query sequence, but not the exact query sequence (e.g., Mo-pterin oxidoreductases versus a specific protein within this family), (ii) sequences that match a query sequence due to homologous regions, but are clearly associated with a gene or gene cluster with different function, and (iii) sequences that returned mis-annotated “blastn” relatives. It is also possible that our inventory of metabolic potential has missed sequences related to a specific query gene. For example, some homologous genes found in the metagenome data were of insufficient length relative to a specific query sequence to make a definitive assignment. Clearly, the metagenomes obtained here do not represent complete sequence for all sub-dominant populations in these sites, thus the functional analysis also cannot be considered complete for these representatives. Phylogenetic analysis was performed on amino acid sequences (aligned in MUSCLE; Edgar, 2004) of select functional genes in MEGA 5 (Tamura et al., 2011) using maximum-likelihood analysis with bootstrapping (1000 replicates).

## Conflict of Interest Statement

The authors declare that the research was conducted in the absence of any commercial or financial `relationships that could be construed as a potential conflict of interest.

## Supplementary Material

The Supplementary Material for this article can be found online at http://www.frontiersin.org/Microbial_Physiology_and_Metabolism/10.3389/fmicb.2013.00084/abstract

Supplementary Table S1 **Number of Aquificales-like single-copy genes identified in assembled metagenome sequence using AMPHORA (Wu and Eisen, 2008)**.

Supplementary Table S2 **TIGRFAM electron transport gene family counts across six Aquificales streamer communities and results for comparison of low-pH and high-pH sites using White’s non-parametric *T*-test**.

Supplementary Table S3 **TIGRFAM functional category gene family counts across six Aquificales streamer communities and results for comparison of taxonomically distinct sites (Hydrogenobaculum-dominated, Sulfurihydrogenibium-dominated, Thermocrinis (Aquificaceae)-dominated) using ANOVA**.
