# Supplementary material for: Metagenome Sequence Analysis of Filamentous Microbial Communities Obtained from Geochemically Distinct Geothermal Channels Reveals Specialization of Three Aquificales Lineages
Source: Front Microbiol. 2013 May 29;4:84. doi: 10.3389/fmicb.2013.00084 (PMC3665934; doi:10.3389/fmicb.2013.00084)
Supplement: Supplementary Table S1 — Number of Aquificales-like single-copy genes identified in assembled metagenome sequence using AMPHORA (Wu and Eisen, 2008). [file 41698_Inskeep_DataSheet1.PDF]

Table S1. Number of Aquificales-like single copy genes identified in assembled metagenome sequence using AMPHORA (Wu and Eisen, 2008).

Gene Name	Dragon (DS_9)	100 Spring Plain (OSP_14)	Mammoth (MHS_10)	Calcite (CS_12)	Bechler (BCH_13)	Octopus (OS_11)
dnaG	0	0	0	2	7	9
frr	5	3	1	7	13	10
infC	2	1	1	4	8	9
nusA	2	2	1	3	4	5
pgk	13	8	1	13	13	19
pyrG	12	15	2	12	20	21
rplA	0	1	1	4	6	9
rplB	5	2	1	4	6	6
rplC	2	1	1	5	4	6
rplD	1	1	1	5	4	5
rplE	2	2	1	4	5	10
rplF	1	2	1	3	5	6
rplK	7	3	1	3	6	9
rplL	1	1	1	4	7	5
rplM	1	2	1	7	6	5
rplN	6	4	1	3	3	10
rplP	0	0	1	4	6	10
rplS	1	2	2	5	7	7
rplT	1	2	1	5	6	6
rpmA	2	4	2	3	6	6
rpoB	1	2	3	17	21	23
rpsB	3	4	1	6	8	10
rpsC	2	2	1	7	9	8
rpsE	2	2	1	3	5	5
rpsI	2	4	1	4	7	13
rpsJ	6	6	1	5	7	9
rpsK	7	3	1	2	7	15
rpsM	6	0	1	3	5	8
rpsS	2	3	1	3	4	10
smpB	2	3	3	7	6	6
tsf	2	3	1	8	13	8
**Average No. single copy genes**	**3**	**3**	**1**	**5**	**8**	**9**

Table S2. TIGRFAM electron transport gene family counts across six Aquificales streamer communities and results for comparison of low pH and high pH sites using White's non-parametric T-test.

Label	p-values	pH 2-5: mean	pH 2-5: std. d	pH 5-9: mean	pH 5-9: std. d	Difference be	95.0% lower	95.0% upper
TIGR00403 NADH-plastoquinone oxidoreductase, I subunit	0.00938333	2.24047367	0.16635863	3.55440836	0.21449987	-1.31393469	-1.61434666	-1.01352272
TIGR01421 glutathione-disulfide reductase	0.01713333	11.2108096	1.15091607	5.18651593	0.71664031	6.02429367	4.39224394	7.67801591
TIGR01553 formate dehydrogenase, alpha subunit	0.02138333	2.37852716	0.13806576	3.89800589	0.51649009	-1.51947873	-2.15009271	-1.06365844
TIGR01292 thioredoxin-disulfide reductase	0.02348333	14.2445817	1.0347325	7.28481537	2.02673346	6.95976629	4.51226559	9.40726698
TIGR00391 hydrogenase (NiFe) small subunit (hydA)	0.03096667	1.92381072	0.47193019	0.04810005	0.08331173	1.87571067	1.35568043	2.39574091
TIGR03148 cytochrome c nitrite reductase, NrfD subunit	0.03416667	0.99301708	0.20485337	0.1490886	0.15196877	0.84392848	0.5332981	1.13373706
TIGR01973 NADH dehydrogenase (quinone), G subunit	0.03601667	1.52444695	0.01725669	3.3987011	0.88476201	-1.87425415	-2.73909949	-0.90017208
TIGR03095 rusticyanin	0.04055	0.65927535	0.16148774	0.0733704	0.12708127	0.58590494	0.3510468	0.82076309
TIGR02699 archaeoflavoprotein AfpA	0.04141667	0.53523947	0.44650832	2.20653467	0.44381374	-1.6712952	-2.39287243	-0.94971798
TIGR00782 cytochrome c oxidase, cbb3-type, subunit III	0.0535	0	0	1.14968684	0.70720609	-1.14968684	-1.83779492	-0.37571691
TIGR01958 NADH-quinone oxidoreductase, E subunit	0.05833333	0.68267892	0.06044441	1.45145558	0.46648065	-0.76877665	-1.25031939	-0.26774484
TIGR01974 proton-translocating NADH-quinone oxidoreductase, chain	0.06696667	12.0014456	1.12078895	8.68633973	0.91679544	3.31510584	1.59526923	5.03494245
TIGR02693 arsenite oxidase, large subunit	0.06995	1.43737829	0.13759953	2.10755138	0.3713	-0.67017309	-1.11948782	-0.29137864
TIGR00561 NAD(P)(+) transhydrogenase (AB-specific), alpha subunit	0.08136667	0	0	0.89693622	0.61825744	-0.89693622	-1.52915763	-0.24050024
TIGR01959 NADH oxidoreductase (quinone), F subunit	0.11508333	1.31004812	0.17619856	1.90505649	0.35429955	-0.59500837	-1.00442522	-0.19730561
TIGR00238 KamA family protein	0.13493333	0.76427551	0.01006078	2.05681614	1.0794335	-1.29254063	-2.43630707	-0.25814149
TIGR00353 cytochrome c-type biogenesis protein CcmF	0.14041667	0.43500591	0.1861121	1.44316178	0.8473482	-1.00815587	-1.73539352	-0.03224772
TIGR01433 ubiquinol oxidase, subunit II	0.14375	0.03881988	0.03881988	0.60401614	0.51628248	-0.56519626	-1.07999421	-0.12151362
TIGR01961 NADH (or F420H2) dehydrogenase, subunit C	0.15788333	2.66150492	0.25553146	4.01237441	1.22308864	-1.35086949	-2.6758981	-0.26513762
TIGR02842 cytochrome o ubiquinol oxidase, subunit III	0.16298333	0.11645963	0.11645963	1.0173976	0.84681926	-0.90093797	-1.78959268	-0.06792389
TIGR01957 NADH-quinone oxidoreductase, B subunit	0.16625	3.65385946	0.03806532	2.80432228	0.81963345	0.84953718	0.01231369	1.57946042
TIGR02897 cytochrome aa3 quinol oxidase, subunit III	0.16838333	0.11645963	0.11645963	1.0637368	0.90058903	-0.94727717	-1.92861028	-0.06792389
TIGR01432 cytochrome aa3 quinol oxidase, subunit II	0.17151667	0.03881988	0.03881988	0.67916375	0.63312452	-0.64034387	-1.30543705	-0.08269374
TIGR03149 cytochrome c nitrite reductase, Fe-S protein	0.17533333	2.90233783	0.19216084	3.5782064	0.5918478	-0.67586857	-1.3304664	-0.00287286
TIGR00320 desulfoferrodoxin	0.17661667	0	0	0.87012294	0.87681806	-0.87012294	-1.81671802	0
TIGR02229 caa(3)-type oxidase, subunit IV	0.18288333	0	0	0.13693322	0.13823094	-0.13693322	-0.27386643	0
TIGR03147 cytochrome c nitrite reductase, accessory protein NrfF	0.19753333	0	0	0.12147045	0.12661853	-0.12147045	-0.24294091	0
TIGR03152 formate-dependent cytochrome c nitrite reductase, c552 su	0.20531667	0	0	0.12919365	0.13736138	-0.12919365	-0.25838731	0
TIGR00397 MauM/NapG family ferredoxin-type protein	0.23805	0	0	0.09370557	0.10773382	-0.09370557	-0.19694161	0
TIGR01582 formate dehydrogenase, beta subunit	0.2557	2.65876888	0.28045029	3.3380414	0.63292417	-0.67927253	-1.35566397	0.09527447
TIGR01755 NAD(P)H:quinone oxidoreductase, type IV	0.27955	0.19964508	0.19964508	0.58537079	0.36796993	-0.38572571	-0.8170668	0.06336161
TIGR01416 ubiquinol-cytochrome c reductase, iron-sulfur subunit	0.29055	1.07209847	0.32541706	1.53241167	0.28339715	-0.4603132	-0.9580539	0.04124682
TIGR01945 electron transport complex, RnfABCDGE type, C subunit	0.29593333	0.98185208	0.13837862	1.46885519	0.64101918	-0.48700311	-1.08977536	0.26969091
TIGR01583 formate dehydrogenase, gamma subunit	0.30201667	0.1608252	0.1608252	0.49931526	0.39599151	-0.33849006	-0.74399438	0.10470848
TIGR01943 electron transport complex, RnfABCDGE type, A subunit	0.36576667	0	0	0.05611672	0.09719702	-0.05611672	-0.16835017	0
TIGR02181 glutaredoxin 3	0.36576667	0	0	0.0270312	0.04681941	-0.0270312	-0.08109361	0
TIGR02657 amicyanin	0.36576667	0	0	0.0270312	0.04681941	-0.0270312	-0.08109361	0
TIGR03094 sulfocyanin	0.36576667	0.43811003	0.43811003	0	0	0.43811003	0	0.87622005
TIGR03872 cytochrome c(L), periplasmic	0.36576667	0	0	0.057924	0.10032732	-0.057924	-0.17377201	0
TIGR03980 hybrid cluster protein-associated redox disulfide domain	0.36576667	0	0	0.0502008	0.08695034	-0.0502008	-0.15060241	0
TIGR03096 nitrosocyanin	0.46878333	0	0	0.08109361	0.14045824	-0.08109361	-0.24328082	0
TIGR00319 desulfoferrodoxin FeS4 iron-binding domain	0.47991667	0	0	0.06012506	0.10413966	-0.06012506	-0.18037518	0

Table S2 (Takacs-Vesbach et al., continued)

TIGR00273 iron-sulfur cluster-binding protein	0.49545	2.12098354	0.14671557	1.84719995	0.41248967	0.27378359	-0.15426542	0.74195165
TIGR01962 NADH dehydrogenase (quinone), D subunit	0.52061667	4.90708259	0.09513569	4.45279398	0.88576416	0.45428861	-0.58974768	1.12420587
TIGR02866 cytochrome c oxidase, subunit II	0.5928	0.69665359	0.15738368	1.08922839	0.93951405	-0.3925748	-1.32146969	0.5363201
TIGR00780 cytochrome c oxidase, cbb3-type, subunit I	0.63545	1.42081481	0.17634579	1.14770951	0.68888969	0.27310531	-0.56718799	0.89101128
TIGR03477 DMSO reductase family type II enzyme, heme b subunit	0.66658333	0.44624453	0.3079702	0.75388116	0.80163216	-0.30763662	-1.28161829	0.47363112
TIGR02694 arsenite oxidase, small subunit	0.69043333	1.53964241	0.00903016	1.31484895	0.76177682	0.22479345	-0.27255139	1.10328158
TIGR01944 electron transport complex, RnfABCDGE type, B subunit	0.70156667	2.8311701	0.22469273	2.56537723	0.83012773	0.26579288	-0.67536137	1.04343424
TIGR01971 NADH-quinone oxidoreductase, chain I	0.71871667	4.94709258	1.05401361	4.36642225	1.07344725	0.58067033	-1.07388389	2.36851335
TIGR01915 NADPH-dependent F420 reductase	0.74323333	0.40949816	0.25421866	0.27477307	0.32229598	0.13472509	-0.31643518	0.58588536
TIGR00129 formate dehydrogenase family accessory protein FdhD	0.77416667	0.26064774	0.26064774	0.15676191	0.14832945	0.10388583	-0.22993914	0.464742
TIGR01813 flavocytochrome c	0.79075	3.41480559	0.71959707	3.71836585	0.74313599	-0.30356026	-1.46899444	0.91664695
TIGR01068 thioredoxin	0.80853333	3.89887005	0.08343083	3.98935126	0.43746477	-0.09048121	-0.44463944	0.40109032
TIGR00276 putative iron-sulfur cluster-binding protein	0.84843333	0.21432522	0.21432522	0.29323164	0.33606886	-0.07890642	-0.49789645	0.34008361
TIGR02891 cytochrome c oxidase, subunit I	0.88366667	2.06966131	0.82519228	2.25411418	0.31900045	-0.18445287	-1.20799285	0.8390871
TIGR02882 cytochrome aa3 quinol oxidase, subunit I	0.91901667	1.27895173	0.73968181	1.15744237	0.96223734	0.12150936	-1.22058209	1.46360081
TIGR01753 flavodoxin	0.95275	0.45630531	0.45630531	0.50209808	0.31204977	-0.04579277	-0.69903969	0.60745415
TIGR00203 cytochrome d ubiquinol oxidase, subunit II	0.99041667	0.81707005	0.12569837	0.82785644	0.87978852	-0.0107864	-0.86627172	0.81707005
TIGR00402 ferredoxin-type protein NapF	0.99356667	0.88960579	0.53009252	0.89783914	0.57998328	-0.00823335	-0.90158584	0.87524875

Table S2 (Takacs-Vesbach et al., continued)

DS_9 Dragon	rel. freq. (%)	OSP_14 100 !	rel. freq. (%)	BCH_13 Becl	rel. freq. (%)	CS_12 Calcit	rel. freq. (%)	MHS_10 Man	rel. freq. (%)	OS_11 Octop	rf. (%)
150	2.07411504	217	2.4068323	315	3.38164251	208	3.33493667	274	3.87115004	235	3.62990423
894	12.3617257	907	10.0598935	569	6.10842727	326	5.22687189	290	4.0972026	344	5.31356194
182	2.51659292	202	2.2404614	327	3.51046699	297	4.76190476	271	3.82876519	226	3.49088662
1105	15.2793142	1191	13.2098492	912	9.79066023	464	7.43947411	293	4.13958745	503	7.7695397
105	1.45188053	216	2.39574091	0	0	12	0.19240019	0	0	0	0
57	0.78816372	108	1.19787045	0	0	16	0.25653359	0	0	22	0.33982082
109	1.50719027	139	1.54170364	422	4.53032743	216	3.46320346	145	2.0486013	230	3.55267223
36	0.49778761	74	0.82076309	0	0	0	0	0	0	19	0.29348162
71	0.98174779	8	0.08873114	240	2.57648953	164	2.6294693	148	2.09098615	99	1.5291937
0	0	0	0	27	0.28985507	105	1.68350168	141	1.99208816	41	0.63330244
45	0.62223451	67	0.74312334	72	0.77294686	91	1.45903479	148	2.09098615	96	1.48285449
949	13.1222345	981	10.8806566	756	8.11594203	485	7.77617444	720	10.1723651	562	8.68087736
94	1.29977876	142	1.57497782	191	2.05045625	170	2.72566939	134	1.89319017	114	1.76088971
0	0	0	0	82	0.88030059	60	0.96200096	0	0	113	1.74544331
82	1.13384956	134	1.48624667	140	1.50295223	149	2.38896906	148	2.09098615	106	1.6373185
56	0.77433628	68	0.75421473	344	3.69296833	110	1.76366843	148	2.09098615	44	0.67964164
18	0.24889381	56	0.62111801	162	1.73913043	135	2.16450216	0	0	121	1.86901452
0	0	7	0.07763975	129	1.38486312	20	0.32066699	0	0	46	0.71053445
174	2.40597345	263	2.91703638	371	3.9828234	216	3.46320346	422	5.9621362	171	2.64133457
0	0	21	0.23291925	162	1.73913043	23	0.36876704	0	0	127	1.96169293
267	3.69192478	326	3.61579414	271	2.9092861	119	1.90796857	289	4.08307431	150	2.31696015
0	0	21	0.23291925	162	1.73913043	23	0.36876704	0	0	139	2.14704974
0	0	7	0.07763975	157	1.68545357	20	0.32066699	0	0	46	0.71053445
196	2.71017699	279	3.09449867	331	3.55340848	264	4.23280423	275	3.88527833	171	2.64133457
0	0	0	0	0	0	99	1.58730159	134	1.89319017	0	0
0	0	0	0	28	0.30059045	0	0	0	0	16	0.24714242
0	0	0	0	0	0	12	0.19240019	0	0	19	0.29348162
0	0	0	0	0	0	12	0.19240019	0	0	21	0.32437442
0	0	0	0	0	0	7	0.11223345	0	0	17	0.26258882
172	2.37831858	265	2.93921917	312	3.34943639	238	3.81593715	275	3.88527833	149	2.30151375
0	0	36	0.39929015	81	0.86956522	34	0.54513388	0	0	60	0.92678406
54	0.74668142	126	1.39751553	118	1.26677402	122	1.95606862	115	1.62475276	83	1.28205128
61	0.84347345	101	1.1202307	52	0.5582394	128	2.05226872	148	2.09098615	76	1.17392648
0	0	29	0.3216504	86	0.92324208	14	0.22446689	0	0	55	0.84955205
0	0	0	0	0	0	14	0.22446689	0	0	0	0
0	0	0	0	0	0	0	0	0	0	7	0.10812481
0	0	0	0	0	0	0	0	0	0	7	0.10812481
0	0	79	0.87622005	0	0	0	0	0	0	0	0
0	0	0	0	0	0	0	0	0	0	15	0.23169601
0	0	0	0	0	0	0	0	0	0	13	0.20080321
0	0	0	0	0	0	0	0	0	0	21	0.32437442
0	0	0	0	0	0	15	0.24050024	0	0	0	0

Table S2 (Takacs-Vesbach et al., continued)

164	2.26769912	178	1.97426797	111	1.19162641	116	1.85986853	143	2.02034473	150	2.31696015
348	4.8119469	451	5.00221828	382	4.10091251	253	4.05643739	422	5.9621362	239	3.69168984
39	0.53926991	77	0.85403727	199	2.13633924	20	0.32066699	0	0	123	1.89990732
90	1.24446903	144	1.5971606	62	0.66559313	73	1.1704345	160	2.26052557	32	0.49428483
10	0.13827434	68	0.75421473	0	0	70	1.12233446	134	1.89319017	0	0
112	1.54867257	138	1.53061224	172	1.84648417	104	1.66746833	0	0	113	1.74544331
221	3.05586283	235	2.60647737	205	2.20075148	149	2.38896906	279	3.94179147	112	1.72999691
434	6.00110619	351	3.89307897	278	2.98443371	244	3.91213725	419	5.91975134	301	4.6493667
48	0.66371681	14	0.1552795	29	0.31132582	0	0	0	0	51	0.78776645
0	0	47	0.52129547	11	0.1180891	25	0.40083373	0	0	7	0.10812481
299	4.13440265	243	2.69520852	248	2.66237252	216	3.46320346	289	4.08307431	302	4.6648131
288	3.98230088	344	3.81543922	302	3.24208266	266	4.26487093	292	4.12545917	280	4.32499228
31	0.42865044	0	0	33	0.35426731	0	0	0	0	53	0.81865925
90	1.24446903	261	2.89485359	216	2.31884058	122	1.95606862	141	1.99208816	178	2.74945938
39	0.53926991	182	2.01863354	162	1.73913043	30	0.48100048	0	0	156	2.40963855
66	0.91261062	0	0	45	0.48309179	46	0.73753407	0	0	51	0.78776645
50	0.69137168	85	0.94276841	0	0	77	1.2345679	147	2.07685787	0	0
26	0.35951327	128	1.41969831	41	0.4401503	38	0.60926728	134	1.89319017	42	0.64874884

Table S3. TIGRFAM functional category gene family counts across six Aquificales streamer communities and results for comparison of taxonomically distinct sites (Hydrogenobaculum-dominated, Sulfurihydrogenibium-dominated, Thermocrinis (Aquificaceae)-dominated) using ANOVA.

**Broad category**	**Specific category**	**p-values**	**Effect size**	**Aquificaceae**	**std. dev. (%)**	**Hydrogenoba**	**std. dev. (%)**
Unclassified	Role category not yet assigned	0.00061978	0.99273069	0.00315758	0.00183038	0.03162428	0.0010455
Biosynthesis of cofactors, prosthetic grour	Pyridine nucleotides	0.0046531	0.97212842	0.23179614	0.00361009	0.14006504	0.0123306
Signal transduction	PTS	0.00567233	0.96819411	0.0218747	0.0032372	0	0
Transcription	Transcription factors	0.0110601	0.95035914	0.26968456	0.01336711	0.20768222	0.04440431
Cellular processes	Detoxification	0.01272568	0.94549278	0.47259931	0.05373207	0.62225839	0.01426941
DNA metabolism	DNA replication, recombination, and repair	0.02975767	0.9039717	3.10477238	0.24812523	2.50241722	0.03870018
Cellular processes	Sporulation and germination	0.03068436	0.90198828	0.79996241	0.10832332	0.55311252	0.10684815
Signal transduction	Two-component systems	0.03129808	0.90068569	0.65511721	0.04094888	0.41285345	0.14033345
Protein synthesis	tRNA and rRNA base modification	0.03719932	0.88856457	1.51119421	0.11930614	1.27449915	0.03663944
Biosynthesis of cofactors, prosthetic grour	Chlorophyll and bacteriochlorphyll	0.03983495	0.88336125	0.35037249	0.04641152	0.40096027	0.02554165
Regulatory functions	Protein interactions	0.04059581	0.88188072	0.24522617	0.0144935	0.2011702	0.03090976
Mobile and extrachromosomal element fur	Prophage functions	0.04114952	0.88080908	0.09146055	0.02915691	0.03842552	0.00109868
Fatty acid and phospholipid metabolism	Degradation	0.0418718	0.87941838	0.1863961	0.04289046	0.19619912	0.01944889
Transport and binding proteins	Cations and iron carrying compounds	0.04675067	0.87022475	2.96320742	0.04331272	3.20913929	0.0068983
Cellular processes	DNA transformation	0.05213395	0.86044446	0.10709835	0.01282713	0.04874694	0.00651246
Energy metabolism	Fermentation	0.06237021	0.84272799	0.35704251	0.04101596	0.30508766	0.020993
Purines, pyrimidines, nucleosides, and nu	Nucleotide and nucleoside interconversions	0.07113971	0.82831142	0.20203849	0.03501346	0.21468824	0.01058857
Purines, pyrimidines, nucleosides, and nu	2'-Deoxyribonucleotide metabolism	0.08024135	0.81396321	0.21265063	0.00476979	0.3067828	0.00552491
Regulatory functions	DNA interactions	0.08605851	0.80507724	0.51298514	0.09633904	0.43938497	0.11430919
Protein fate	Protein and peptide secretion and trafficking	0.09187517	0.79639016	2.65026789	0.10481288	2.28172211	0.03544058
DNA metabolism	Degradation of DNA	0.09211782	0.79603182	0.03757558	0.00803788	0.00537327	0.0027136
Biosynthesis of cofactors, prosthetic grour	Folic acid	0.10820081	0.77293318	0.20082854	0.03316079	0.16065145	0.00634899
Energy metabolism	Amino acids and amines	0.1129792	0.76629626	1.22872906	0.06069356	1.2177925	0.12543888
Cell envelope	Biosynthesis and degradation of surface polysaccharides and	0.11412918	0.76471307	1.71851685	0.1051391	1.37489768	0.11991686
Transcription	Degradation of RNA	0.11901783	0.75804125	0.22869537	0.03202827	0.17220932	0.0027971
Not defined	Not defined	0.13496006	0.73689031	24.7028827	1.19364935	26.2929617	0.18888708
Energy metabolism	Sugars	0.13758342	0.7334917	0.47887166	0.01894222	0.55065286	0.00358422
Cellular processes	Cell division	0.16094355	0.70411992	0.77211188	0.04414731	0.66007988	0.00227461
Cellular processes	Toxin production and resistance	0.16385558	0.70056161	0.07105124	0.00640304	0.09801454	0.01716416
Energy metabolism	Glycolysis/gluconeogenesis	0.17073848	0.69223379	0.44597756	0.04069718	0.38556909	0.05695076
Signal transduction	Other	0.17406131	0.68825356	10.4165159	0.58806693	10.2493797	0.32589216
Cellular processes	Adaptations to atypical conditions	0.17599592	0.68594788	0.42866941	0.06140845	0.53693652	0.03807046
Unknown function	General	0.1782096	0.68331993	1.59237274	0.18797633	1.66756335	0.05176618
Energy metabolism	Photosynthesis	0.17963814	0.68162983	0.02330628	0.0045978	0.02336556	0.0051326
Energy metabolism	TCA cycle	0.18030236	0.68084552	0.8842042	0.02723752	0.88309249	0.01387608
Central intermediary metabolism	Nitrogen fixation	0.18572464	0.67447849	0.14421786	0.0289694	0.12415089	0.01212458
Biosynthesis of cofactors, prosthetic grour	Lipoate	0.19929744	0.65880619	0.05028173	0.01509872	0.01580586	0.00052761
Cellular processes	Conjugation	0.20700352	0.6500668	0.08223303	0.00189109	0.09311287	0.00229058
Cell envelope	Biosynthesis and degradation of murein sacculus and peptido(	0.2131929	0.6431258	1.24472797	0.27005731	0.81872501	0.14299344
Transport and binding proteins	Carbohydrates, organic alcohols, and acids	0.21651402	0.6394291	1.87957055	0.31129101	2.37411442	0.20564684
Amino acid biosynthesis	Serine family	0.21977256	0.63582037	0.40463932	0.01948871	0.54257141	0.09922566
Biosynthesis of cofactors, prosthetic grour	Pantothenate and coenzyme A	0.2199117	0.63566669	0.2579875	0.0580537	0.17327694	0.02767699

Table S3., Takacs-Vesbach et al., continued.

Energy metabolism	Entner-Doudoroff	0.22318325	0.6320622	0.02662117	0.00379268	0.01691804	0.00306473
Protein synthesis	Ribosomal proteins: synthesis and modification	0.22430718	0.63082797	1.38904484	0.00515776	1.26159291	0.11263121
Amino acid biosynthesis	Aromatic amino acid family	0.25587586	0.59695561	0.62495064	0.13096012	0.61636894	0.01648717
Transport and binding proteins	Nucleosides, purines and pyrimidines	0.25590091	0.5969293	0.00643775	0.00315912	0.01861287	0.00987774
Amino acid biosynthesis	Histidine family	0.25659256	0.59620335	0.40519066	0.05280016	0.39856589	0.03809391
Transport and binding proteins	Unknown substrate	0.26554186	0.58686816	1.39899487	0.10082271	1.22222213	0.04597457
Energy metabolism	Methanogenesis	0.26730907	0.58503723	0.64284319	0.06752453	0.62363384	0.0371582
Energy metabolism	Biosynthesis and degradation of polysaccharides	0.28295325	0.5690008	0.71700863	0.03951414	0.65969384	0.03125185
Energy metabolism	Anaerobic	0.28511423	0.56680916	0.13305356	0.04014651	0.16425722	0.00260222
Amino acid biosynthesis	Glutamate family	0.30991024	0.54204379	0.57188195	0.0512524	0.67994899	0.01863674
Amino acid biosynthesis	Pyruvate family	0.31504822	0.53699606	0.73463436	0.06832323	0.77405116	0.00351684
Biosynthesis of cofactors, prosthetic grou	Molybdopterin	0.3206395	0.53153406	0.30636863	0.00701451	0.4532977	0.09694256
Unknown function	Enzymes of unknown specificity	0.32299559	0.52924198	2.00310503	0.25469105	2.18210194	0.21651845
Transcription	RNA processing	0.32717022	0.52519439	0.22756196	0.05973674	0.21742862	0.01518181
Biosynthesis of cofactors, prosthetic grou	Biotin	0.33194098	0.52058982	0.31301104	0.06065612	0.28470904	0.01172959
Protein fate	Protein modification and repair	0.33354879	0.51904301	1.09177172	0.14441944	1.2507867	0.00803812
Amino acid biosynthesis	Aspartate family	0.35634612	0.49737044	0.84310021	0.03516526	0.89887	0.06776657
Fatty acid and phospholipid metabolism	Biosynthesis	0.36282948	0.49129222	0.85830248	0.16543105	0.627004	0.11536546
Transport and binding proteins	Porins	0.38573946	0.47009741	0.28469585	0.12091372	0.23303544	0.0382977
Transport and binding proteins	Amino acids, peptides and amines	0.39465401	0.46196439	3.79963695	0.72941207	3.67026413	0.38577383
Energy metabolism	ATP-proton motive force interconversion	0.40217814	0.45514747	0.50009952	0.08958215	0.48215255	0.03798733
Transport and binding proteins	Anions	0.40681097	0.45097123	2.83176257	0.37520043	2.50165515	0.17684327
Cellular processes	Pathogenesis	0.40975542	0.44832521	1.24934727	0.054368	1.02960641	0.08078151
Biosynthesis of cofactors, prosthetic grou	Riboflavin, FMN, and FAD	0.41729723	0.44157652	0.19859508	0.05194485	0.1537246	0.02216663
Protein fate	Degradation of proteins, peptides, and glycopeptides	0.42779947	0.43224606	0.81333554	0.04349014	0.84274492	0.06333419
Biosynthesis of cofactors, prosthetic grou	Glutathione and analogs	0.43378404	0.42696338	0.26505155	0.04718079	0.28264224	0.03198568
Purines, pyrimidines, nucleosides, and nu	Salvage of nucleosides and nucleotides	0.4388572	0.42250421	0.14309762	0.01327949	0.20653178	0.04895137
Purines, pyrimidines, nucleosides, and nu	Purine ribonucleotide biosynthesis	0.44600522	0.41625036	1.01422099	0.14349899	1.05372407	0.13517021
DNA metabolism	Chromosome-associated proteins	0.45151257	0.4114547	0.15831801	0.02307027	0.19696383	0.02510321
Biosynthesis of cofactors, prosthetic grou	Heme, porphyrin, and cobalamin	0.45492706	0.40849124	0.85966933	0.12425563	1.01132008	0.03851149
Regulatory functions	RNA interactions	0.464758	0.4	0.00160131	0.00160131	0	0
Central intermediary metabolism	Sulfur metabolism	0.47910802	0.38771217	0.30712188	0.02554752	0.31588457	0.06920943
Mobile and extrachromosomal element fu	Transposon functions	0.48768746	0.38042428	0.35968606	0.22332852	0.89256072	0.2683152
Energy metabolism	Electron transport	0.49411693	0.37499068	1.84157563	0.34123985	2.05597787	0.22463276
Protein synthesis	Translation factors	0.50526383	0.36562586	1.19086295	0.11564766	1.26853809	0.00366963
Central intermediary metabolism	Polyamine biosynthesis	0.51627472	0.35644267	0.25040823	0.03408962	0.23467354	0.01171511
Cellular processes	Biosynthesis of natural products	0.51962592	0.35366074	1.24733012	0.15112799	1.16898263	0.05495232
Energy metabolism	Pyruvate dehydrogenase	0.53239149	0.34311794	0.12757593	0.03629592	0.09199638	0.00348582
Energy metabolism	Aerobic	0.55626536	0.32362428	0.03170355	0.00217938	0.04237867	0.01688292
Central intermediary metabolism	Amino sugars	0.56460622	0.31687984	0.33267209	0.02239183	0.35657704	0.02355708
Biosynthesis of cofactors, prosthetic grou	Menaquinone and ubiquinone	0.5679703	0.31416905	0.85468669	0.02275683	0.87375304	0.02488267
Central intermediary metabolism	Phosphorus compounds	0.59721722	0.29082261	0.44455081	0.07003241	0.40979067	0.02440814
Biosynthesis of cofactors, prosthetic grou	Pyridoxine	0.63630127	0.26020988	0.12733267	0.01579953	0.10458978	0.02547586
Protein fate	Protein folding and stabilization	0.63768446	0.25913816	0.4194776	0.09480101	0.33422816	0.00215703

Table S3., Takacs-Vesbach et al., continued.

Regulatory functions	Small molecule interactions	0.64711437	0.25185227	0.06962935	0.01547782	0.11646653	0.04406771
Hypothetical proteins	Domain	0.64877843	0.25057024	0.00065645	0.00065645	0.00070979	0.00070979
Central intermediary metabolism	One-carbon metabolism	0.65915328	0.24260178	0.0629768	0.00269471	0.06439405	0.01763567
Central intermediary metabolism	Nitrogen metabolism	0.66723286	0.23642515	0.30946583	0.06547543	0.30059906	0.04721506
Transcription	DNA-dependent RNA polymerase	0.70037187	0.21134729	0.24704268	0.05069053	0.20074751	0.01312418
Energy metabolism	Pentose phosphate pathway	0.74612792	0.17736182	0.27678195	0.04005191	0.27102724	0.02944851
Cellular processes	Chemotaxis and motility	0.76114331	0.16636177	0.54013688	0.0221423	0.73287205	0.14175507
Cell envelope	Surface structures	0.79033067	0.14518417	0.10300692	0.04247396	0.07273048	0.00714749
Energy metabolism	Chemoautotrophy	0.82941494	0.11722932	0.00305841	0.00037949	0.00691394	0.00691394
DNA metabolism	Restriction/modification	0.86777494	0.09021647	0.14924244	0.03733943	0.15473781	0.01898886
Biosynthesis of cofactors, prosthetic grou	Thiamine	0.8843282	0.07868322	0.40306486	0.03378051	0.38626823	0.03153869
Mobile and extrachromosomal element fu	Plasmid functions	0.88975296	0.07491929	0.12458574	0.00021284	0.12395048	0.02569872
Purines, pyrimidines, nucleosides, and nu	Pyrimidine ribonucleotide biosynthesis	0.91049773	0.06059563	0.47648071	0.06076298	0.47091412	0.0116317
Protein synthesis	tRNA aminoacylation	0.92016908	0.05395508	2.3600729	0.23998939	2.44986396	0.03040406
Hypothetical proteins	Conserved	0.99494461	0.00337311	0.68262842	0.04975343	0.67632787	0.03073165

Table S3., Takacs-Vesbach et al., continued.

Sulfurihydro	std. dev. (%	BCH_13 Be	rel. freq. (	OS_11 Octop	rel. freq. (	DS_9 Dragon	rel. freq. (%	OSP_14 100	rel. freq. (	CS_12 Calc	rel. freq. (%	MHS_10 Ma	rl. frq. (%)
0	0	7.25	0.001327	27.24	0.004988	166.94	0.030579	178.4	0.03267	0	0	0	0
0.24614769	0.004986	1285.94	0.235406	1246.16	0.228186	831.98	0.152396	697.52	0.127734	1371.69	0.251133	1317.15	0.2411619
0	0	101.81	0.018638	137.14	0.025112	0	0	0	0	0	0	0	0
0.60972372	0.052383	1546.21	0.283052	1399.79	0.256317	891.39	0.163278	1376.57	0.252087	3044.19	0.55734	3616.22	0.66210719
0.2819961	0.016206	2875.16	0.526331	2287.5	0.418867	3319.22	0.607989	3475.89	0.636528	1628.78	0.298202	1451.66	0.26578984
3.62984671	0.067497	18315.68	3.352898	15600.6	2.856647	13872.83	2.541117	13453.63	2.463717	19457.54	3.56235	20193.72	3.69734343
1.54689442	0.187179	4961.64	0.908286	3777.15	0.691639	2436.31	0.446264	3603.85	0.659961	7426.76	1.359715	9470.96	1.73407335
1.17232078	0.108715	3802.36	0.696066	3354.07	0.614168	1487.78	0.27252	3020.79	0.553187	6997.01	1.281035	5809.08	1.0636061
2.0090588	0.140299	8906.84	1.6305	7601.32	1.391888	6757.9	1.23786	7159.74	1.311139	10207.16	1.86876	11739.11	2.14935739
0.15133608	0.042337	1660.43	0.303961	2166.9	0.396784	2328.42	0.426502	2050.05	0.375419	1057.84	0.193673	595.32	0.10899936
0.38459764	0.035925	1260.41	0.230733	1418.37	0.25972	929.51	0.17026	1267.32	0.23208	1904.45	0.348673	2296.76	0.42052235
0.15822201	0.011132	658.89	0.120617	340.25	0.062304	203.78	0.037327	215.83	0.039524	925.01	0.169354	803.36	0.14709018
0.02119281	0.020827	783.92	0.143506	1252.17	0.229287	964.94	0.17675	1177.59	0.215648	229.51	0.042019	2	0.00036619
2.71941074	0.126331	15950.34	2.919895	16419.08	3.00652	17482.13	3.202241	17561.83	3.216038	15543.43	2.845742	14162.58	2.59307954
0.08401907	0.008555	655.11	0.119925	514.83	0.094271	301.68	0.055259	230.63	0.042234	505.64	0.092574	412.16	0.07546391
0.20842886	0.000136	1726.34	0.316027	2173.86	0.398058	1550.97	0.284095	1780.63	0.326081	1139.18	0.208565	1137.63	0.20829292
0.30801822	0.00713	1294.93	0.237052	912.15	0.167025	1114.25	0.2041	1230.17	0.225277	1643.45	0.300888	1721.24	0.31514824
0.22323334	0.03409	1187.69	0.21742	1135.27	0.207881	1644.67	0.301258	1705.42	0.312308	1405.5	0.257324	1033.04	0.18914314
0.84370322	0.010475	3328.52	0.609324	2275.37	0.416646	1774.7	0.325076	3023.56	0.553694	4551.09	0.833228	4665.25	0.854178
2.43071192	0.07303	13904.91	2.545455	15045.93	2.755081	12263.22	2.246282	12653.34	2.317163	13675.43	2.503742	12876.91	2.35768143
0.03680508	0.010051	249.17	0.045613	161.31	0.029538	14.52	0.00266	44.16	0.008087	255.93	0.046857	146.12	0.02675366
0.24947991	0.004751	1278.2	0.233989	915.66	0.167668	842.39	0.154302	911.94	0.167	1388.61	0.254231	1336.63	0.24472857
0.91416467	0.004979	6380.56	1.168035	7041.74	1.289423	7333.16	1.343231	5965.02	1.092354	4965.97	0.909186	5020.07	0.91914332
1.89603807	0.133272	9961.98	1.823656	8810.91	1.613378	6851.37	1.254981	8162.74	1.494815	9628.22	1.762766	11083.45	2.02931016
0.26468959	0.018806	1424.24	0.260724	1074.03	0.196667	955.42	0.175006	925.11	0.169412	1548.45	0.283495	1342.94	0.24588389
23.2687033	0.416896	128422.53	23.50923	141425.04	25.89653	144573.48	26.48185	142546.63	26.10407	129370.64	23.6856	124809.34	22.8518071
0.45514777	0.037753	2512.43	0.459929	2718.64	0.497814	3025.77	0.554237	2987.38	0.547069	2692.22	0.4929	2279.68	0.41739511
0.6768287	0.03339	3976.61	0.727965	4457.72	0.816259	3616.02	0.662354	3592.08	0.657805	3879.21	0.710218	3514.26	0.64343896
0.04364231	0.017211	353.15	0.064648	422.99	0.077454	628.8	0.115179	441.5	0.08085	332.38	0.060853	144.36	0.02643141
0.54709955	0.031991	2658.53	0.486675	2213.3	0.40528	1794.04	0.328618	2416.47	0.44252	2813.52	0.515108	3162.81	0.57909067
9.09582002	0.128283	53689.3	9.828449	60097.76	11.00458	57734.03	10.57527	54189.23	9.923488	50382.01	9.224103	48977.85	8.96753704
0.38431433	0.020583	2677.12	0.490078	2005.67	0.367261	3139.16	0.575007	2724.16	0.498866	1986.7	0.363732	2211.42	0.40489713
2.0528983	0.136233	9725.41	1.780349	7669.63	1.404396	8821.19	1.615797	9388.75	1.71933	10468.82	1.916666	11956.34	2.18913084
0.00643721	0.006437	152.43	0.027904	102.17	0.018708	99.54	0.018233	155.62	0.028498	70.32	0.012874	0	0
0.94675478	0.017618	4978.88	0.911442	4680.03	0.856967	4745.35	0.869216	4898.08	0.896969	5074.94	0.929137	5267.1	0.96437296
0.18679974	0.001292	946.06	0.173187	629.39	0.115248	611.59	0.112026	744.16	0.136275	1013.24	0.185507	1027.3	0.18809218
0.0531208	0.014789	357.15	0.06538	192.14	0.035183	89.17	0.016333	83.43	0.015278	209.37	0.038332	370.9	0.06790946
0.06790533	0.012778	459.54	0.084124	438.76	0.080342	495.83	0.090822	520.97	0.095403	440.69	0.080683	301.09	0.05512769
1.38970217	0.06618	8274.73	1.514785	5322.83	0.974671	3689.05	0.675732	5251.66	0.961718	7229.07	1.323522	7951.57	1.45588258
1.67941016	0.070879	8566.95	1.56828	11964.64	2.190862	14083.8	2.579761	11841.36	2.168468	9560.07	1.750289	8785.29	1.60853148
0.35952853	0.013864	2103.94	0.385151	2316.23	0.424128	3503.79	0.641797	2420.98	0.443346	1888.02	0.345665	2039.35	0.37339219
0.29571868	0.019259	1726.42	0.316041	1091.87	0.199934	794.88	0.1456	1097.35	0.200954	1510.02	0.276459	1720.31	0.31497797

Table S3., Takacs-Vesbach et al., continued.

0.02428676	0.002467	166.14	0.030414	124.67	0.022828	75.63	0.013853	109.12	0.019983	119.18	0.02182	146.12	0.02675366
1.75174389	0.250966	7559.68	1.383887	7613.96	1.394203	7502.36	1.374224	6274.14	1.148962	8197.24	1.500777	10938.17	2.0027103
0.82459135	0.036356	4129.27	0.755911	2697.76	0.493991	3274.96	0.599882	3455.84	0.632856	4305.34	0.788236	4702.22	0.86094698
0.00110399	0.001104	17.91	0.003279	52.41	0.009597	155.54	0.028491	47.7	0.008735	12.06	0.002208	0	0
0.50185515	0.016999	2501.84	0.457991	1924.46	0.35239	1967.94	0.360472	2384.47	0.43666	2833.98	0.518854	2648.13	0.48485599
1.23174227	0.040485	7091.45	1.298172	8190.74	1.499818	6923.52	1.268197	6423.14	1.176248	6948.9	1.272227	6506.27	1.19125722
0.52173423	0.008352	3142.76	0.575319	3879.43	0.710368	3607.49	0.660792	3202.57	0.586476	2895.33	0.530086	2803.93	0.51338199
0.76558939	0.041457	3700.91	0.677494	4131.49	0.756523	3430.88	0.628442	3773.05	0.690946	3955.21	0.724133	4407.83	0.80704602
0.1989826	0.006637	946.13	0.1732	507.38	0.092907	882.53	0.161655	911.17	0.166859	1050.59	0.192345	1123.03	0.20561975
0.63093247	0.044423	3403.96	0.623134	2843.24	0.52063	3610.33	0.661312	3814.77	0.698586	3203.51	0.586509	3688.58	0.67535586
0.8378752	0.00043	4386.27	0.802958	3638.83	0.666311	4245.01	0.777568	4207.66	0.770534	4578.82	0.838305	4573.86	0.83744507
0.40437716	0.020499	1711.9	0.313383	1634.82	0.299354	3003.95	0.55024	1945.95	0.356355	2096.74	0.383878	2320.54	0.42487631
1.68693008	0.009585	9550.96	1.748414	12330.18	2.257796	13094.89	2.39862	10733.47	1.965583	9161.65	1.677345	9265.83	1.69651534
0.30328282	0.013457	1569.41	0.287299	916.52	0.167825	1269.9	0.23261	1104.41	0.202247	1583.03	0.289826	1729.93	0.31673933
0.40654424	0.06059	2041.21	0.373667	1378.15	0.252355	1618.36	0.296439	1490.66	0.272979	1889.6	0.345954	2551.34	0.46713435
1.35818202	0.111235	6752.87	1.236191	5173.64	0.947352	6784.59	1.242749	6874.07	1.258825	6810.82	1.246947	8025.49	1.46941687
0.95361879	0.018521	4797.65	0.878265	4412.26	0.807935	5277.2	0.966637	4538.41	0.831103	5107.5	0.935098	5309.52	0.9721398
0.88484196	0.031262	5592.29	1.023734	3783.88	0.692871	2793.21	0.511639	4053.86	0.742369	4662.25	0.85358	5003.47	0.91610396
0.41202667	0.055188	2215.7	0.40561	894.44	0.163782	1063.14	0.194738	1481.67	0.271333	1949.05	0.356838	2551.78	0.46721491
2.75713394	0.266951	16771.54	3.070225	24733.85	4.529049	22143.3	4.056038	17935.63	3.28449	16517.54	3.024085	13600.59	2.4901827
0.60136182	0.020561	3221.22	0.589682	2241.9	0.410517	2424.85	0.444165	2840.33	0.52014	3396.94	0.621923	3172.15	0.58080076
2.26967151	0.149087	13419.32	2.456562	17513.73	3.206963	14622.84	2.678498	12695.11	2.324812	13211.25	2.418759	11581.96	2.12058421
1.03599407	0.170329	7121.73	1.303715	6525.97	1.194979	5179.96	0.948825	6063.5	1.110388	6588.93	1.206323	4727.99	0.86566531
0.23188417	0.026483	1368.61	0.25054	800.88	0.14665	718.22	0.131558	960.49	0.175891	1121.9	0.205401	1411.12	0.25836722
0.92183183	0.048667	4205.39	0.769845	4679.26	0.856826	4255.07	0.779411	4947.83	0.906079	4769.22	0.873164	5300.56	0.97049928
0.34971316	0.045918	1190.15	0.217871	1705.15	0.312232	1368.42	0.250657	1718.09	0.314628	1659.33	0.303796	2160.81	0.39563075
0.17402981	0.013341	709.15	0.129818	854	0.156377	1394.77	0.255483	860.5	0.15758	1023.42	0.187371	877.63	0.16068855
1.26062029	0.101282	6324.21	1.15772	4755.15	0.870722	5014.7	0.918554	6492.2	1.188894	6332.3	1.159338	7438.28	1.3619024
0.20441944	0.024281	738.81	0.135248	990.59	0.181388	1212.34	0.222067	938.48	0.171861	1249.16	0.2287	983.86	0.18013859
0.91135271	0.017057	5374.83	0.983925	4016.21	0.735414	5731.39	1.049832	5312.22	0.972809	5070.97	0.92841	4884.36	0.89429567
0	0	0	0	17.49	0.003203	0	0	0	0	0	0	0	0
0.23749655	0.019604	1538.14	0.281574	1816.76	0.332669	2102.36	0.385094	1347.02	0.246675	1190.13	0.217893	1404.2	0.25710021
0.64675889	0.331398	3184.8	0.583015	744.67	0.136358	3407.97	0.624246	6339.2	1.160876	5342.69	0.978157	1722.4	0.31536063
1.58829721	0.125672	11923.94	2.182815	8193.57	1.500336	9997.94	1.831345	12453.74	2.280611	7988.85	1.462625	9361.16	1.71396966
1.55234644	0.33505	7137	1.306511	5871.92	1.075215	6905.35	1.264868	6947.15	1.272208	6648.87	1.217297	10308.36	1.88739604
0.29350039	0.045276	1554.11	0.284498	1181.35	0.216319	1345.12	0.246389	1217.51	0.222958	1355.8	0.248224	1850.29	0.33877649
1.07200282	0.048329	5988.16	1.096202	7637.2	1.398458	6681.88	1.223935	6083.39	1.11403	6119.25	1.120332	5590.98	1.02367336
0.09838189	0.006943	498.63	0.09128	894.93	0.163872	521.27	0.095482	483.33	0.088511	499.44	0.091439	575.25	0.10532467
0.0259519	0.000802	161.28	0.029524	185.04	0.033883	139.19	0.025496	323.61	0.059262	137.37	0.02515	146.12	0.02675366
0.38418221	0.042527	1694.95	0.31028	1939.06	0.355064	1818.07	0.33302	2075.8	0.380134	1866.12	0.341655	2330.55	0.42670908
0.82667465	0.036211	4793.16	0.877444	4543.3	0.83193	4905.96	0.898636	4635.43	0.84887	4713.08	0.862886	4317.26	0.79046322
0.37520637	0.019045	2045.86	0.374518	2810.22	0.514583	2370.44	0.434199	2104.46	0.385383	2153.4	0.394252	1945.24	0.35616124
0.10658319	0.001821	781.88	0.143132	609.1	0.111533	431.91	0.079114	710.25	0.130066	572.21	0.104762	592.07	0.1084043
0.43625577	0.090124	2809.32	0.514279	1773.11	0.324677	1812.89	0.332071	1836.9	0.336385	1890.57	0.346132	2874.92	0.52637981

Table S3., Takacs-Vesbach et al., continued.

0.10085895	0.034607	464.91	0.085107	295.73	0.054152	395.25	0.072399	876.63	0.160534	361.87	0.066252	739.87	0.13546555
0	0	0	0	7.17	0.001313	7.75	0.00142	0	0	0	0	0	0
0.0513225	0.001723	358.74	0.065672	329.21	0.060282	255.27	0.046758	447.94	0.08203	270.91	0.049599	289.72	0.05304591
0.24990995	0.012364	2048.17	0.374941	1332.47	0.24399	1383.31	0.253384	1899.31	0.347814	1432.54	0.262274	1297.4	0.2375458
0.25801473	0.064444	1626.41	0.297733	1072.31	0.196352	1024.3	0.187623	1167.89	0.213872	1057.28	0.19357	1761.17	0.32245918
0.24593714	0.004902	1730.75	0.316834	1292.82	0.23673	1640.4	0.300476	1319.19	0.241579	1370.08	0.250839	1316.46	0.24103557
0.76654191	0.359133	3071.53	0.562279	2828.85	0.517995	3227.11	0.591117	4776.08	0.874627	6148.43	1.125675	2225.14	0.40740917
0.10384108	0.042951	794.71	0.145481	330.58	0.060533	358.04	0.065583	436.19	0.079878	801.78	0.146793	332.56	0.06088965
0.00861956	0.00862	18.78	0.003438	14.63	0.002679	0	0	75.51	0.013828	94.16	0.017239	0	0
0.17307341	0.037227	1019.23	0.186582	611.12	0.111903	741.1	0.135749	948.67	0.173727	1148.66	0.2103	741.95	0.13584639
0.38631237	0.007793	2386.33	0.436845	2016.72	0.369284	1936.59	0.35473	2281.52	0.417807	2067.47	0.378519	2152.48	0.39410558
0.13678927	0.025152	681.73	0.124799	679.22	0.124373	536.39	0.098252	817.19	0.149649	884.52	0.161941	609.73	0.11163774
0.49289113	0.014841	2934.77	0.537244	2270.3	0.415718	2507.38	0.459282	2635.04	0.482546	2611.11	0.478051	2773.07	0.50773172
2.38594154	0.128045	14203.21	2.600062	11578.11	2.120084	13208.66	2.41946	13544.01	2.480268	12332.62	2.257896	13730.6	2.51398671
0.67818327	0.052677	4000.74	0.732382	3456.23	0.632875	3860.08	0.70706	3525.41	0.645596	3416.51	0.625506	3991.73	0.73086072
